# Nicotinamide Mononucleotide Adenylyltransferase 1 and NAD^+^ Homeostasis in Neuroprotection and Aging

**DOI:** 10.3390/metabo16080597

**Published:** 2026-08-21

**Authors:** You Sun, Bowei Li, Zhengjiang Qian

**Affiliations:** 1 Department of Biomedical Engineering, Southern University of Science and Technology, Shenzhen 518055, China; 2 Faculty of Life and Health Sciences, Shenzhen University of Advanced Technology (SUAT), Shenzhen 518107, China; 3 Brain Cognition and Brain Disease Institute (BCBDI), Shenzhen Institutes of Advanced Technology (SIAT), Chinese Academy of Sciences, Shenzhen 518055, China

**Keywords:** NMNAT-1, NAD^+^, neuroprotection, aging, axon degeneration, SARM1, sirtuins, PARP1, proteostasis, retinal degeneration

## Abstract

Nicotinamide adenine dinucleotide (NAD^+^) is a fundamental metabolic cofactor and signaling molecule that supports redox reactions, DNA repair, chromatin regulation, stress adaptation, inflammation, and neuronal maintenance. Age-associated NAD^+^ decline has been implicated in brain aging and neurodegenerative disorders, but the causal node and limiting compartment differ across tissues and disease states. Nicotinamide mononucleotide adenylyltransferase 1 (NMNAT-1) catalyzes the final step in NAD^+^ biosynthesis and represents the major nuclear isoform of the mammalian NMNAT family. Direct human genetic evidence establishes NMNAT-1 as a causal gene in inherited retinal degeneration, whereas evidence linking endogenous NMNAT-1 to broader brain aging or sporadic neurodegeneration is mainly convergent preclinical, preliminary, or indirect. Beyond NAD^+^ synthesis, biochemical and Drosophila studies suggest possible chaperone-like and proteostasis-supporting functions, but a separable NAD^+^-independent function of endogenous mammalian NMNAT-1 has not yet been established in vivo. Here, we review the molecular structure, localization, and regulation of NMNAT-1, emphasizing calibrated distinctions among catalytic nuclear NAD^+^ supply, engineered axonal protection, pathway-adjacent NAD^+^ interventions, and putative non-catalytic protection. We further discuss how NMNAT-1 dysfunction may contribute to aging-associated genomic instability, neuroinflammation, synaptic impairment, retinal degeneration, selected neurodegenerative models, and glioma biology. Finally, we evaluate therapeutic strategies targeting NMNAT-1 and NAD^+^ pathways, noting that no human trial has yet established efficacy for an NMNAT-1-directed neurological therapy. A compartment-aware and evidence-stratified view is therefore essential for translating NMNAT-1 biology into interventions for age-related neural disease.

## 1. Introduction

The nervous system is unusually sensitive to disturbances in nicotinamide adenine dinucleotide (NAD^+^) metabolism [1,2]. Neurons must sustain long axons, polarized trafficking, electrical activity, synaptic transmission, and proteome maintenance for decades, while differentiated neurons have limited capacity to dilute damaged macromolecules by cell division. NAD^+^ supports these demands in two conceptually distinct ways. First, the NAD^+^/NADH couple transfers reducing equivalents through glycolysis, the tricarboxylic acid cycle, oxidative phosphorylation, and biosynthetic reactions [3,4]. Second, oxidized NAD^+^ is cleaved by sirtuins, poly(ADP-ribose) polymerases (PARPs), CD38-family enzymes, and sterile alpha and TIR motif-containing protein 1 (SARM1) [5,6]. These reactions connect NAD^+^ availability to transcription, DNA repair, inflammation, calcium signaling, mitochondrial quality control, and programmed axon destruction. Because NAD^+^ is consumed rather than merely recycled in the second class of reactions, its concentration reflects a dynamic balance among precursor delivery, synthesis, compartmental exchange, and degradation.

Interest in this balance has expanded rapidly as NAD^+^ abundance was found to decline in multiple tissues with age and as NAD^+^ augmentation improved selected physiological outcomes in diverse animal models [7,8]. Yet “NAD^+^ decline” is not a single mechanism. It can arise from reduced precursor availability, impaired salvage, increased PARP or CD38 activity, changes in cell composition, or redistribution among subcellular pools [5,9]. Nor does an increase in whole-tissue NAD^+^ prove restoration of the nuclear, cytosolic, mitochondrial, or axonal pool that limits a particular phenotype. These distinctions are especially important in the brain, where neurons and glia have different metabolic programs and where local axonal degeneration can proceed despite an apparently viable cell body [1,5].

Nicotinamide mononucleotide adenylyltransferases (NMNATs) occupy a strategic position in this network. They catalyze adenylyl transfer from ATP to nicotinamide mononucleotide (NMN), generating NAD^+^, and can also use nicotinic acid mononucleotide (NaMN) to generate nicotinic acid adenine dinucleotide [10,11]. Mammals express three NMNAT isoforms with different targeting information and tissue distributions [12,13]. NMNAT-1 is predominantly nuclear, NMNAT-2 is associated with the cytosolic face of Golgi-derived vesicles and is actively transported into axons, and NMNAT-3 has classically been assigned to mitochondria, although tissue- and model-dependent localization remains under discussion [12,14]. NMNAT-1 is therefore positioned not merely at the final step of NAD^+^ synthesis but at a nuclear decision point where locally produced NAD^+^ can be delivered to chromatin-associated consumers [15,16].

The neuroprotective reputation of NMNAT-1 arises from several partially overlapping studies. The Wld^S^ mutation produces a chimeric UBE4B–NMNAT-1 protein that dramatically delays Wallerian degeneration [17,18]. NMNAT-1 overexpression, particularly when the protein is redirected to axons or the cytoplasm, can suppress SARM1-dependent NAD^+^ collapse [19,20]. Drosophila Nmnat additionally protects against activity stress and misfolded proteins even when catalytic activity is impaired, motivating the concept that NMNAT proteins can act as molecular chaperones [21,22]. Finally, biallelic NMNAT-1 variants cause Leber congenital amaurosis type 9 (LCA9) and related retinal phenotypes, providing direct human evidence that NMNAT-1 is indispensable for neural tissue integrity [23,24]. These bodies of work are often discussed together, but they do not answer the same question. Wld^S^ and axon-targeted constructs test whether NMNAT-1 activity can be engineered to protect a threatened axon; retinal genetics tests what endogenous nuclear NMNAT-1 is required to do; and chaperone studies test whether NMNAT proteins possess functions not reducible to net NAD^+^ synthesis.

This review develops a unified but evidence-stratified account of NMNAT-1 in neuroprotection and aging. We distinguish direct NMNAT-1 evidence from results obtained with other NMNAT isoforms, WldS, NAD^+^ precursors, or downstream NAD^+^ consumers. We also distinguish catalytic dependence from simple correlations with total NAD^+^ and from proposed non-catalytic activity. Our central framework is that NMNAT-1 function is governed by three linked axes: compartment, catalytic flux relative to demand, and cell state, with molecular partnerships determining local coupling to PARP1, sirtuins, SARM1, or client proteins. The same enzyme may support homeostasis by supplying basal nuclear NAD^+^, promote repair by coupling to PARP1, influence chromatin through sirtuins, or protect an axon only after artificial relocalization. Conversely, increasing NAD^+^ can be ineffective or harmful when delivery misses the limiting compartment, when downstream damage is irreversible, or when it supports unwanted cell survival. This framework guides the discussion of mechanism, aging, disease, and therapeutic translation that follows.

### Evidence-Grading and Terminology Boundaries

Because the NMNAT-1 literature combines human genetics, engineered axon-protection constructs, NAD^+^ precursor studies, tumor models, and ortholog experiments, we assign evidence grades to specific claims rather than to whole disease categories. Strong/direct evidence denotes replicated human genetics or endogenous mammalian loss/rescue with a concordant biochemical mechanism. Moderate/convergent preclinical evidence denotes reproducible mammalian in vivo or multi-model intervention data with a plausible mechanism but limited human causal validation. Preliminary evidence denotes single-model, overexpression, in vitro, or ortholog findings that generate testable hypotheses. Indirect/pathway-adjacent evidence denotes studies of NMNAT-2, WldS, SARM1, CD38/PARP demand, or systemic NAD^+^ precursors that inform the pathway but do not directly test endogenous NMNAT-1.

Terminology is used accordingly. In this review, “NMNAT-1” refers to endogenous or full-length mammalian NMNAT-1 unless otherwise specified; “WldS” refers to the chimeric UBE4B-NMNAT-1 protein; and “axon-targeted NMNAT-1” refers to engineered constructs redirected to cytoplasmic or axonal compartments. WldS and axon-targeted NMNAT-1 demonstrate engineered axonal sufficiency, but they are not treated as proof that endogenous nuclear NMNAT-1 normally maintains distal axons.

## 2. Molecular and Biological Characteristics of NMNAT-1

### 2.1. The Mammalian NMNAT Family

NMNAT enzymes catalyze a chemically conserved reaction but are not biologically interchangeable. Human NMNAT-1 was initially characterized as a nuclear enzyme essential for NAD^+^ synthesis, and structural studies established a compact α/β phosphotransferase fold organized around a conserved ATP-binding region and a nucleotide-binding pocket [10,25]. Mammalian isoforms share catalytic logic while differing in N- or C-terminal targeting sequences, oligomeric behavior, kinetic properties, protein stability, and partner interactions [12,26]. These differences partition NAD^+^ biosynthetic capacity across the cell and explain why an isoform that produces NAD^+^ efficiently in vitro may fail to rescue loss of another isoform in vivo.

NMNAT-1 is a soluble nuclear protein containing a nuclear localization signal [10,26]. It is broadly expressed and is more stable than the short-lived neuronal survival factor NMNAT-2. NMNAT-2 associates with Golgi membranes through palmitoylation-dependent targeting and travels in vesicular carriers toward distal axons [14,27]. Its rapid turnover makes continued axonal delivery necessary; injury or transport failure therefore lowers NMNAT-2 before many other proteins, allowing the NMN/NAD^+^ ratio to rise and SARM1 to activate [28,29]. NMNAT-3 was identified as a third human isoform and is often described as mitochondrial. However, endogenous localization and physiological contribution vary across tissues, and the discovery of the SLC25A51 mitochondrial NAD^+^ transporter has shown that mammalian mitochondria can import intact NAD^+^, reducing the need to assume that all mitochondrial NAD^+^ is synthesized in situ [30,31].

The family comparison carries an important interpretive warning. Nuclear NMNAT-1 is not the constitutive axonal survival factor in healthy mammalian neurons; NMNAT-2 fills that role [28,32]. Conversely, a fall in NMNAT-2 or activation of SARM1 does not establish that endogenous NMNAT-1 was defective. NMNAT-1 becomes relevant to axons when it is expressed at high levels, fused to localization motifs, released into cytoplasm in particular constructs, or incorporated into Wld^S^ [19,33]. Throughout this review, “NMNAT-1-mediated axon protection” therefore refers to a demonstrated intervention, whereas “physiological axon maintenance” is attributed primarily to the NMNAT-2–SARM1 axis unless direct evidence indicates otherwise.

### 2.2. Catalytic Reaction, Structure, and Substrate Handling

NMNAT-1 transfers the adenylyl moiety of ATP to NMN to form NAD^+^ and pyrophosphate [10,11]. With NaMN as the substrate, it forms nicotinic acid adenine dinucleotide, which is subsequently amidated to NAD^+^ in the Preiss–Handler and de novo pathways [10,11]. Divalent cations facilitate catalysis, and the enzyme can operate reversibly under appropriate in vitro conditions, although cellular substrate/product ratios favor biosynthesis. Structural analyses show that ATP and NMN/NaMN occupy an active-site cleft whose geometry supports dual substrate specificity [10,25]. Because ATP is consumed and pyrophosphate hydrolysis can pull the reaction forward, NMNAT-1 activity is also sensitive to the energetic and ionic environment.

It is tempting to label NMNAT-1 “rate limiting,” but this is not universally correct. NAMPT often controls flux through the nicotinamide salvage pathway, while precursor delivery or NAD^+^ consumption can dominate under other conditions [34,35]. NMNAT-1 becomes limiting when its abundance, localization, or catalytic capacity is insufficient relative to the supply of NMN/NaMN and the demand from nuclear consumers. This conditional control is evident in pathogenic NMNAT-1 variants, in which catalytic impairment or protein instability can markedly affect the retina, and in cancer models, in which high nuclear NAD^+^ demand creates dependence on NMNAT-1 [36,37]. Thus, flux control is distributed across the pathway rather than permanently assigned to one enzyme.

The active site may also participate in proposed chaperone activity. Biochemical work on phosphorylated tau indicates that an NAD^+^ substrate-binding surface can engage amyloidogenic clients [38]. Consequently, mutations used to abolish catalysis may alter client binding, conformation, oligomerization, or stability [38,39]. Experiments claiming enzyme-independent protection must therefore verify not only loss of catalytic activity but also protein abundance, folding, localization, oligomeric state, and residual NAD^+^ production under cellular conditions. Catalytic and chaperone functions may share structural elements and need not behave as separable on/off modules.

### 2.3. Tissue Expression and Subcellular Localization

Expression surveys indicate that NMNAT-1 is broadly and relatively low-specifically expressed across CNS and non-CNS tissues, including nervous, cardiovascular, skeletal-muscle, hepatic, pancreatic, adipose, renal, gastrointestinal, endocrine, and immune compartments [40,41]. Broad expression, however, does not imply uniform dependence. Nuclear NAD^+^ demand differs with transcriptional activity, DNA damage burden, chromatin state, developmental stage, and cell identity. Neurons continuously repair oxidative DNA lesions and maintain activity-dependent transcription; retinal photoreceptors combine high metabolic flux, light-induced oxidative stress, outer-segment renewal, and specialized developmental programs [42,43]. These features can convert a modest biochemical defect into severe tissue-selective pathology.

Within the CNS, NMNAT-1 should not be treated as neuron-only. Available datasets and mechanistic considerations support expression or potential dependence in neuronal, astrocytic, oligodendroglial, microglial, endothelial, vascular, and choroid-plexus compartments, although cell-specific functional evidence remains much stronger for retinal progenitors and photoreceptors than for most glial or vascular populations. This distinction is important for aging studies because bulk tissue measurements can average together cells with very different NAD^+^ precursor supply, consumption, inflammatory state, and repair demand.

Nuclear localization provides proximity to PARP1, SIRT1, SIRT6, SIRT7, and other chromatin-associated NAD^+^ consumers [44,45]. Proximity matters because NAD^+^ can be locally produced and consumed faster than bulk diffusion and whole-cell assays reveal. Metabolite sensors have documented distinct subcellular NAD^+^ dynamics, while compartment-specific manipulations show that local synthesis can regulate transcription without proportionate changes in total cellular NAD^+^ [15,46]. NMNAT-1 can therefore influence a nuclear process even when whole-cell NAD^+^ appears only modestly altered.

Subcellular targeting is equally decisive for neuroprotection. Nuclear sequestration limits access of NMNAT-1 to the distal axon, whereas axonal targeting greatly increases potency [19]. In this sense, endogenous NMNAT-1 and engineered NMNAT-1 are the same catalyst operating in different reaction neighborhoods. Their phenotypes differ because substrate levels, competing consumers, diffusion distances, and the proximity of SARM1 differ [20,29].

### 2.4. Whole-Body NAD^+^ Homeostasis and Aging Context

Aging is a systemic process, and NAD^+^ remodeling in the brain cannot be interpreted in isolation from peripheral metabolism. Age-related NAD^+^ dysregulation has been linked to altered precursor supply or salvage, increased CD38/PARP demand, inflammation, mitochondrial dysfunction, vascular dysfunction, and tissue-composition changes across muscle, adipose tissue, liver, pancreas, kidney, cardiovascular, immune, and neural compartments [5,8,9,47,48,49,50,51,52,53,54,55,56,57,58]. These shared mechanisms create an important backdrop for CNS aging, but they do not prove that NMNAT-1 itself is the limiting enzyme in every organ.

Two examples illustrate this distinction. Adipocyte-specific Nmnat1 deletion markedly reduced nuclear NAD^+^ in brown adipose tissue but did not impair the tested thermogenic or whole-body metabolic endpoints, indicating that nuclear target engagement can be biochemically strong yet physiologically buffered [59]. Conversely, SHILCA syndrome caused by NMNAT-1 disruption affects multiple tissues, including skeletal, auditory, intellectual-developmental, and retinal phenotypes [60]. Together, these observations support a tissue-threshold model: whole-body NAD^+^ decline, precursor exchange, and local NMNAT-1 reserve interact, but broad expression should not be equated with uniform organ-level dependence.

### 2.5. Regulation of NMNAT-1 Abundance and Activity

Compared with the extensive regulation mapped for NAMPT and NMNAT-2, the control of NMNAT-1 in neural cells remains incompletely defined [27,61]. Its broad basal expression is consistent with a housekeeping requirement, yet there is demand for nuclear NAD^+^ changes over circadian time, differentiation, neuronal activity, DNA damage, hypoxia, and inflammation [61,62]. Regulation can occur at several levels: transcription and alternative promoter use; translation; nuclear import; oligomerization; post-translational modification; binding to chromatin or partner proteins; and proteasomal turnover. The functional consequence of each level depends on whether it changes catalytic flux, localization, or coupling to a consumer.

Phosphorylation provides a direct example because it alters NMNAT-1-dependent regulation of PARP1 [44]. Stress-linked expression and modification of NMNAT family proteins suggest further control by heat-shock, hypoxic, and metabolic pathways [62,63]. These findings motivate neural studies that measure activity rather than mRNA alone. An unchanged transcript can coexist with loss of active protein through destabilization, defective nuclear import, oxidation, or altered oligomerization. Conversely, a modest increase in expression can have a large effect if it occurs at a DNA lesion or promoter where NAD^+^ demand is concentrated.

Substrate and product concentrations also regulate effective activity. NMNAT-1 requires ATP as well as NMN or NaMN, so severe energetic failure may restrict NAD^+^ synthesis even when the enzyme and precursor are abundant [10,11]. Nicotinamide generated by sirtuins and PARPs must be recycled through NAMPT, while pyrophosphate handling influences reaction direction [11,45]. Thus, “NMNAT-1 activity” in vivo is a pathway property. Isotope tracing with labeled nicotinamide, NMN, or NR can quantify flux through the terminal reaction; steady-state NAD^+^ concentration cannot distinguish higher synthesis from lower consumption [15,35].

An additional question is whether NMNAT-1 is mobilized between soluble nucleoplasm, chromatin, and nucleoli. Its involvement in promoter-associated SIRT1 and PARP1 activity and in rRNA transcription implies regulated recruitment [64,65]. Activity-dependent neuronal transcription may create transient NAD^+^ demand at immediate-early genes, while DNA damage may redirect NMNAT-1 toward repair complexes. Mapping this redistribution in live neurons would help explain how a broadly expressed enzyme produces locus- and stimulus-specific effects.

**Figure 1:**
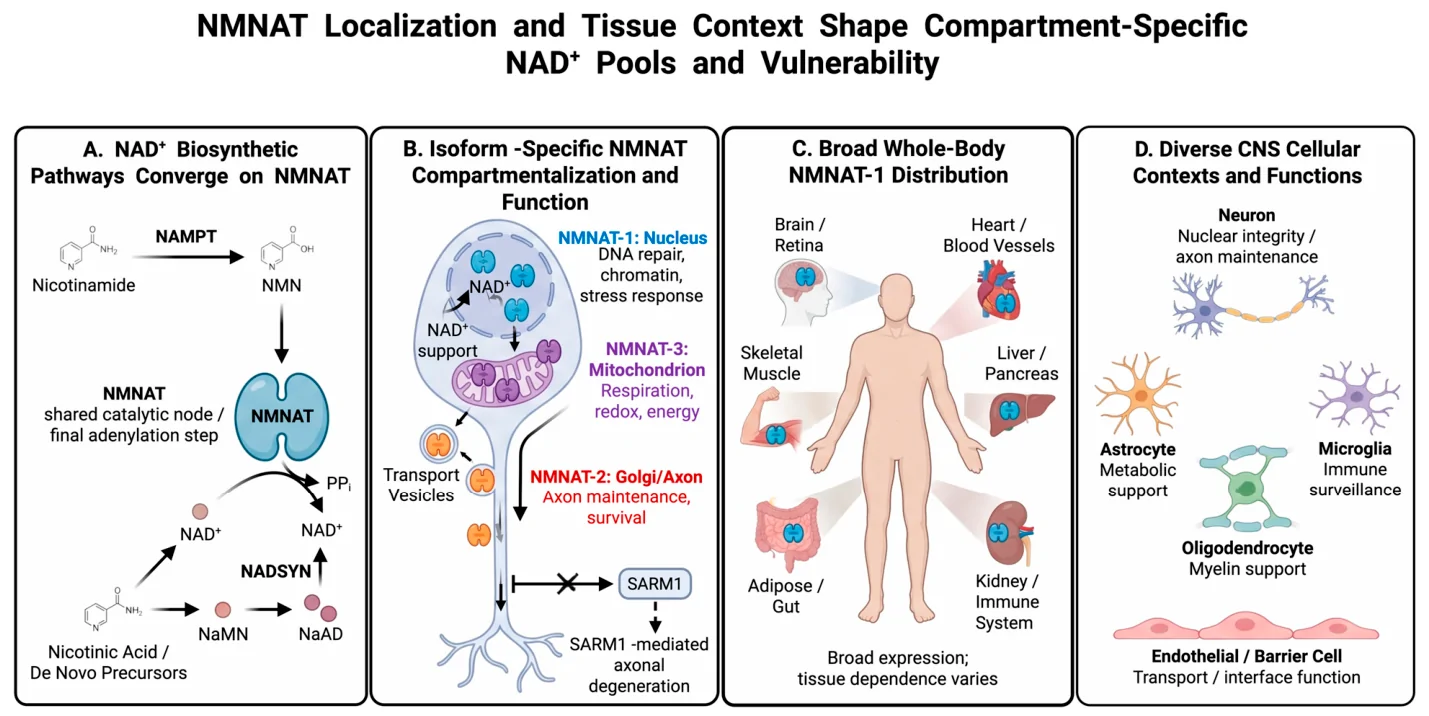
NMNAT localization and tissue context shape compartment-specific NAD+ pools and vulnerability. (**A**) Major NAD+ biosynthetic inputs converge on NMNAT as a shared catalytic node for the final adenylylation step. Nicotinamide-derived NMN and nicotinic-acid/de novo-derived NaMN feed NMNAT-dependent production of NAD+ or NaAD; NADSYN then converts NaAD to NAD+. (**B**) NMNAT isoforms support distinct compartments and functions: NMNAT-1 sustains nuclear NAD+ for DNA repair, chromatin regulation, and stress responses; NMNAT-2 in Golgi/transport vesicles and axons supports axon maintenance and survival; and NMNAT-3 is linked to mitochondrial respiration, redox balance, and energy metabolism. Loss of axonal NAD+ support can permit SARM1-mediated axonal degeneration. (**C**) NMNAT-1 is broadly distributed across brain/retina, heart/blood vessels, skeletal muscle, liver/pancreas, adipose/gut, kidney, and immune compartments, but tissue dependence varies. (**D**) In the CNS, neurons, astrocytes, oligodendrocytes, microglia, and endothelial/barrier cells contribute distinct nuclear-integrity, metabolic-support, myelin-support, immune-surveillance, and transport/interface functions. Thus, localization, tissue context, age, and cell state together determine compartment-specific NAD+ vulnerability.

The pathway, isoform localization, tissue distribution, and CNS cell-context framework used throughout this review are summarized in Figure 1, emphasizing that NMNAT localization and tissue context shape compartment-specific NAD+ pools and vulnerability.

## 3. NMNAT-1 as a Regulator of Nuclear NAD^+^ Homeostasis

### 3.1. NMNAT-1 in Convergent NAD^+^ Biosynthetic Pathways

Mammalian NAD^+^ is synthesized through routes that converge at NMNAT-catalyzed adenylylation. In the dominant nicotinamide salvage pathway, NAMPT converts nicotinamide to NMN, and NMNAT converts NMN to NAD^+^ [29,66]. Nicotinamide riboside (NR) is phosphorylated by NR kinases to NMN, while nicotinic acid enters the Preiss–Handler pathway through NAPRT to form NaMN [34,35]. Tryptophan-derived de novo synthesis also produces NaMN after a multistep kynurenine pathway [34,35]. NMNAT-1 can therefore receive carbon–nitrogen units from several precursors, but the flux reaching the nucleus depends on transporter expression, precursor phosphorylation, cell type, and competition among compartments.

NAMPT and NMNAT-1 form a nuclear salvage module capable of sustaining NAD^+^-dependent enzymes [64,67]. NAMPT-dependent NAD^+^ synthesis regulates mammalian Sir2 homologs, and promoter-associated salvage enzymes can support SIRT1 activity in a spatially restricted manner. Circadian transcription further modulates NAMPT and NAD^+^ abundance, creating feedback between the molecular clock and SIRT1 [61]. These findings support a view of nuclear NAD^+^ synthesis as organized metabolism rather than passive equilibration with a single cellular pool.

The last-step position of NMNAT-1 creates both buffering and vulnerability. Multiple precursors can feed the reaction, which buffers fluctuations upstream. At the same time, a severe NMNAT-1 defect cannot be bypassed simply by supplying more NMN if the nucleus lacks adequate alternative NMNAT activity [34,37]. Indeed, raising NMN may be unhelpful in a compartment where adenylylation is limiting, and, in injured axons, an increased NMN/NAD^+^ ratio can activate SARM1 [29,66]. Precursor therapy must therefore be matched to residual NMNAT capacity and to the compartment in which the pathological bottleneck lies.

### 3.2. Nuclear NAD^+^ Microdomains and Metabolic Coupling

NAD^+^ is small and diffusible, but diffusion does not eliminate spatial regulation. Enzymes can be recruited to promoters, enhancers, DNA lesions, nucleoli, and multiprotein complexes, producing microdomains in which synthesis and consumption are kinetically coupled [15,16]. NMNAT-1 physically and functionally interacts with PARP1. Its phosphorylation state affects PARP1 activity, and promoter-directed recruitment of NMNAT-1 can provide NAD^+^ for PARP1-dependent gene regulation [16,44]. NMNAT-1 also regulates ribosomal RNA transcription, linking nuclear NAD^+^ metabolism to nucleolar biosynthetic capacity [65]. In genotoxic settings, NMNAT-1 expression can favor cell survival, as shown in actinomycin D-treated osteosarcoma cells and acute myeloid leukemia stem cells [36,68]. These cancer studies are not direct evidence for neuroprotection, but they demonstrate a general principle: cells with intense nuclear NAD^+^ demand can become selectively dependent on NMNAT-1.

NMNAT-1 itself is regulated by cell state. Recent work has described lactylation-dependent stabilization or activation of NMNAT-1 in glucose-deprived pancreatic cancer cells, connecting lactate availability to nuclear NAD^+^ salvage [63]. Whether analogous post-translational control operates in neurons or glia remains unknown, but the finding expands the plausible regulatory repertoire beyond transcription and abundance. For neural aging, it suggests that altered glycolytic–lactate coupling could affect nuclear NAD^+^ through modification of NMNAT-1, a hypothesis that requires direct testing in brain tissue.

Compartmentalization is relative rather than absolute. Genetically encoded sensors reveal subcellular NAD^+^ dynamics, and identification of SLC25A51/MCART1 as a mammalian mitochondrial NAD^+^ transporter established a route for direct mitochondrial import [31,69]. Nuclear and cytosolic spaces are not separated by an impermeable membrane, but chromatin binding, local synthesis, and high consumption can still generate functionally distinct nuclear dynamics. Mitochondria, by contrast, maintain a membrane-bounded pool influenced by SLC25A51 and redox shuttles. Accordingly, changing NMNAT-1 may alter mitochondrial function indirectly through transcription, sirtuin signaling, substrate redistribution, or nuclear–mitochondrial communication, without NMNAT-1 being a mitochondrial enzyme.

### 3.3. Coupling to PARPs, Sirtuins, and Chromatin Regulation

PARP1 detects DNA lesions and uses NAD^+^ to build ADP-ribose polymers that reorganize chromatin and recruit repair factors [70,71]. Controlled PARP1 activation supports genome maintenance; excessive activation can rapidly consume NAD^+^, impair energetics, and promote parthanatos or necrotic-like death [70,72]. PARP1 also regulates transcription outside overt DNA damage. NMNAT-1 thus faces a dual task: supplying NAD^+^ for productive repair while preventing the nuclear pool from collapsing under sustained PARP activation. More NMNAT-1 is not automatically protective if damage continues unchecked, because greater NAD^+^ supply can sustain prolonged PARylation. The outcome depends on whether repair resolves the lesion before ATP and NAD^+^ costs become catastrophic.

Sirtuins consume NAD^+^ during deacylation and ADP-ribosylation reactions. Nuclear SIRT1 and SIRT6 regulate stress-responsive transcription, DNA repair, chromatin organization, metabolic adaptation, and inflammatory pathways; SIRT7 contributes to nucleolar and genome functions [45]. Their activities are influenced by NAD^+^ availability, nicotinamide inhibition, substrate recruitment, and protein regulation [61,67]. NMNAT-1 can therefore alter sirtuin function locally without acting as a classical signaling scaffold. This coupling provides plausible routes from NMNAT-1 to antioxidant defenses, mitochondrial biogenesis, autophagy, NF-κB repression, and synaptic plasticity, but each route should be experimentally traced rather than inferred from a rise in total NAD^+^ alone.

PARPs and sirtuins also compete for a shared substrate. During chronic DNA damage, elevated PARP activity can lower NAD^+^ and restrain sirtuin signaling; conversely, PARP inhibition can preserve NAD^+^ but may compromise repair or alter transcription [45,70]. An optimal state is therefore not maximal sirtuin activity or minimal PARP activity, but balanced, lesion-appropriate flux.

### 3.4. An Integrated Compartment-Flux-State Model

An integrated model links the NMNAT-1 phenotype to the match among compartment, catalytic flux, and cell state. Compartment distinguishes endogenous nuclear NMNAT-1 from engineered cytoplasmic or axonal NMNAT-1 and WldS. Flux must be interpreted relative to basal, stress, and reserve demand rather than as total protein abundance or total NAD^+^ alone. Cell state includes age, energy availability, inflammation, development, proteotoxic stress, DNA damage, and tumor transformation, each of which can shift whether NMNAT-1 is protective, neutral, or potentially supportive of unwanted cell survival.

The model generates testable predictions. Nuclear NAD^+^ sensors should reveal deficits before total tissue NAD^+^ falls; stress paradigms should unmask partial-loss variants that appear silent under basal culture conditions; and restoring catalysis in the correct cell type and developmental window should outperform untargeted precursor supplementation when NMNAT-1 capacity is limiting. Conversely, SARM1 inhibition, CD38/PARP modulation, or proteostasis-directed strategies should advance only when biomarkers show that the matched pathway, not generic NAD^+^ abundance, is the limiting defect.

### 3.5. Cell-Type Context in the Nervous System

Neural and neurovascular cell types are unlikely to experience identical NAD+ pressures. During aging, CD38-positive immune populations expand and can lower tissue NAD+ and alter NMN availability [47,48]. Neurons, astrocytes, oligodendrocytes, microglia, endothelial cells, and retinal support cells also differ in energetic demand, stress exposure, and access to circulating precursors; however, direct comparative evidence for cell-type-resolved NAD+ flux remains limited. A bulk brain measurement averages these states and can conceal a deficit in a rare but vulnerable population.

Cell autonomy should therefore be tested directly. In the retina, developmental loss-of-function studies and photoreceptor-targeted rescue support a substantial cell-autonomous requirement for NMNAT-1 [42,73]. Whether comparable requirements apply to neurons, glia, and vascular cells elsewhere in the nervous system remains unresolved. Conditional cell-type-specific deletion, rescue, and co-culture studies are needed to distinguish neuronal from non-cell-autonomous support. Mosaic rescue is particularly informative: if NMNAT-1 expression only in photoreceptors preserves those cells despite a mutant environment, the dominant need is cell autonomous; if neighboring untransduced cells also improve, extracellular or circuit-level mechanisms contribute [73]. Single-cell metabolomics remains technically difficult, but fluorescence sensors, spatial metabolomics, and cell-type-resolved isotope tracing are beginning to close this gap.

Developmental stage is another form of cell state. Progenitors use NAD^+^ for proliferation and lineage-specific chromatin programs, whereas mature neurons use it for maintenance and stress resistance [1,5]. An intervention that rescues adult energetic stress may not restore a developmental transcriptional trajectory already missed. This distinction is central to LCA9 and should inform treatment windows in other NMNAT-1-associated syndromes [42,74].

## 4. NAD^+^-Dependent Mechanisms of NMNAT-1-Mediated Neuroprotection and Aging

### 4.1. From Wld^S^ to a Compartment-Aware Model of Axon Protection

The slow Wallerian degeneration phenotype transformed the study of axon death. Wld^S^ mice carry a tandem triplication that generates a chimeric protein containing the N-terminal portion of UBE4B fused to full-length NMNAT-1 [17,18]. Injured axons in these mice survive far longer than wild-type axons, demonstrating that Wallerian degeneration is an active, genetically controllable program rather than passive decay [17,18]. Early work linked protection to increased nuclear NAD^+^ synthesis and SIRT1, while subsequent studies established that local axonal mechanisms and subcellular targeting are critical [75,76].

NMNAT-1 alone does not reproduce every feature of Wld^S^ under all expression and localization conditions. Nuclear NMNAT-1 was unable to substitute for Wld^S^ in some paradigms, whereas the short Wld^S^ N-terminal sequence, interaction with valosin-containing protein, and NMNAT catalytic activity were required for full protection [33,77]. Transgenic NMNAT-1 nevertheless delayed axonal degeneration in vivo, and targeting NMNAT-1 to axons and synapses markedly increased its potency [19,78]. Direct delivery of NMNAT protein into transected axons was also protective [79]. Together, these findings resolved an apparent contradiction: NMNAT-1 possesses the relevant catalytic capacity, but localization and molecular context determine whether that capacity reaches the degenerating axonal compartment.

The physiological counterpart of this engineered protection is NMNAT-2. Endogenous NMNAT-2 is a labile axon survival factor required for maintenance of healthy axons and embryonic axon integrity [28,32]. Its membrane association and trafficking are regulated by palmitoylation, while ubiquitin-dependent pathways control its abundance [27,80]. Axotomy interrupts delivery, NMNAT-2 falls, and the balance between NMN and NAD^+^ shifts. NMNAT-1 can compensate when deliberately placed in the axon because it is more stable and can restore adenylyltransferase capacity, but this compensation should not be interpreted as evidence that endogenous nuclear NMNAT-1 normally patrols distal axons [19,20].

SARM1 is the executioner that converts loss of NMNAT-2 into rapid metabolic catastrophe. Genetic screens identified dSarm/Sarm1 as essential for injury-induced axon death [81,82]. Activated SARM1 cleaves NAD^+^ locally, and its TIR domain has intrinsic NADase activity [6,83]. Axonal NMNAT-1 blocks this process by maintaining NAD^+^ and limiting SARM1 activation, while SARM1 deletion rescues NMNAT-2-deficient axons [20,84]. Structural and biochemical studies now support a metabolite-sensing mechanism: an increased NMN/NAD^+^ ratio relieves autoinhibition of SARM1, whereas NAD^+^ stabilizes the inactive state [29,66]. Downstream, MAP kinase signaling, energetic failure, and late calcium influx coordinate cytoskeletal breakdown [85,86]. This NMNAT-2–NMN/NAD^+^–SARM1 axis is the best-established catalytic framework for programmed axon degeneration.

### 4.2. Cellular Bioenergetics and Redox Balance

NMNAT-1-generated NAD^+^ can support neuroprotection beyond the axonal SARM1 switch. NAD^+^ is required for glycolytic oxidation of glyceraldehyde-3-phosphate and for multiple mitochondrial redox reactions after reducing equivalents are transferred or NAD^+^ is imported. Depletion restricts ATP production, disrupts ion gradients, impairs vesicle recycling, and increases susceptibility to excitotoxicity [1,3]. Nuclear NAD^+^ additionally supports transcriptional programs that control mitochondrial biogenesis and antioxidant defense. Thus, NMNAT-1 can influence mitochondrial performance indirectly through SIRT1–PGC-1α signaling, DNA repair, or chromatin regulation, even when the protein remains nuclear [45].

Redox protection must be described carefully. NMNAT-1 synthesizes oxidized NAD^+^; it does not directly detoxify reactive oxygen species [1,3]. Its effects depend on downstream metabolism that generates NADH for energy or supports NADPH-producing networks, on sirtuin-dependent transcription, and on prevention of catastrophic NAD^+^ loss. Moreover, the NAD^+^/NADH ratio and total NAD(H) pool convey different information [1,3]. An intervention can raise total NAD^+^ yet fail to correct a highly reduced redox state, and tissue measurements can obscure opposing changes in neurons and glia. Future studies should combine compartment-specific NAD^+^/NADH sensors with ATP, redox, and flux measurements rather than treating a bulk NAD^+^ increase as a complete mechanism.

### 4.3. DNA Repair, Genome Stability, and Cell-Death Thresholds

Post-mitotic neurons accumulate single- and double-strand DNA lesions from oxidative metabolism, activity-associated transcription, and environmental stress [70,72]. Moderate PARP activation promotes repair, whereas sustained PARP1 activity can consume enough NAD^+^ to inhibit glycolysis and mitochondrial function [70,72]. NMNAT-1 may raise the threshold at which repair becomes metabolic collapse by replenishing nuclear NAD^+^ close to PARP1. It may also support SIRT6- and SIRT1-dependent repair pathways and chromatin accessibility [45]. The expected result is not simply less DNA damage at one time point but faster lesion resolution with preserved energy.

This logic also explains why NMNAT-1 can have opposite implications in different diseases. In vulnerable neurons, sufficient nuclear NAD^+^ may permit repair and survival. In malignant cells, the same capacity can support resistance to genotoxic therapy [36,68]. Therapeutic activation of NMNAT-1 in the nervous system must therefore be spatially targeted and monitored for proliferative risk, especially in older individuals with clonal or neoplastic disease. A neuroprotective enzyme is not intrinsically tumor suppressive.

PARP-dependent death and SARM1-dependent axon degeneration both consume NAD^+^, but they differ in trigger, compartment, kinetics, and output. PARP1 is chiefly nuclear and responds to DNA damage; SARM1 is locally activated in axons by metabolic imbalance and injury signals [29,70]. NMNAT-1 can oppose both when present in the relevant compartment, but the necessary intervention differs. Nuclear NMNAT-1 augmentation may improve repair, whereas axonal protection generally requires cytoplasmic/axonal NMNAT activity or direct SARM1 inhibition [20,87]. Collapsing these pathways into a generic “NAD^+^ depletion” mechanism loses therapeutically decisive information.

### 4.4. Sirtuin Signaling, Autophagy, and Inflammatory Control

SIRT1 links nuclear NAD^+^ to deacetylation of transcription factors and coactivators involved in mitochondrial biogenesis, antioxidant responses, autophagy, and inflammatory signaling [45]. SIRT6 supports chromatin stability and DNA repair, while other sirtuins coordinate mitochondrial and stress programs [45,67]. NMNAT-1 can plausibly reinforce these pathways by preserving nuclear NAD^+^, but causal attribution requires rescue or epistasis experiments. For example, loss of protection after SIRT1 inhibition strengthens a specific NMNAT-1–NAD^+^–SIRT1 model; a parallel increase in SIRT1 expression does not.

Autophagy provides an additional bridge between NMNAT activity and proteostasis. In a Drosophila amyloid model, human NMNAT-1 reduced amyloid plaque accumulation by promoting autophagic clearance [88], while Drosophila Nmnat acted upstream of autophagy to limit hypoxia-induced dendrite degeneration [89]. NMNAT-1 has also been associated with AMPK activation and autophagy in ischemic models discussed later [90]. These findings support a context-dependent link between NMNAT proteins and autophagic protection, but they do not establish a universal SIRT1–AMPK–mTOR mechanism. Future studies should measure autophagic flux, lysosomal completion, and cargo removal rather than infer pathway activation from static LC3 or p62 abundance alone.

NAD^+^ metabolism also shapes neuroinflammation. Microglia activated during aging or injury can express high CD38, lowering tissue NAD^+^ and changing extracellular precursor availability [9,48]. SIRT1 can restrain NF-κB-dependent transcription, whereas PARP1 can facilitate inflammatory gene expression [45,91]. NMNAT-1 might therefore influence glial phenotype, but direct cell-type-specific evidence remains limited. Neuron-selective and microglia-selective manipulations are needed to determine whether NMNAT-1 primarily protects the neuronal target, reprograms the inflammatory environment, or does both.

### 4.5. A Mechanistic Hierarchy for Interpreting Protection

To prevent overinterpretation, NMNAT-1 neuroprotection can be assessed at four levels. Level 1 is association: NMNAT-1 expression and survival change together. Level 2 is necessity or sufficiency: genetic loss worsens injury, or targeted expression improves it. Level 3 identifies the biochemical requirement through catalytically inactive variants, compartment-specific constructs, and measurements of local NAD^+^ flux [92,93]. Level 4 establishes the downstream effector through epistasis with PARP1, sirtuins, SARM1, AMPK, or autophagy genes. Many studies reach Levels 1 or 2; fewer reach Levels 3 and 4.

### 4.6. Soma, Axon, Synapse, and Glia as Linked but Separable Therapeutic Units

Neuroprotection is often scored as cell-body survival, yet a living soma with disconnected axons can remain functionally useless. Conversely, an anatomically preserved axon may not conduct or release transmitter normally. NMNAT-1 studies should therefore measure at least three outcomes: soma survival, axonal continuity and transport, and synaptic or circuit function. Wld^S^ illustrates the distinction particularly well because it can preserve injured axons for prolonged periods without correcting the initiating lesion [17,94]. The therapeutic goal is durable function, not delayed fragmentation alone.

The soma and axon also compete and cooperate metabolically. Nuclear NMNAT-1 supports transcription and repair programs needed for the whole neuron, while axonal NMNAT-2 continuously suppresses SARM1 [16,28]. A disease can begin in either compartment and propagate. Nuclear DNA damage may reduce expression or transport of NMNAT-2; distal mitochondrial failure may send stress signals to the nucleus; and glial inflammation can accelerate both. A combined model should therefore measure temporal order rather than assuming that the compartment with the largest terminal pathology was the initiating site.

**Figure 2:**
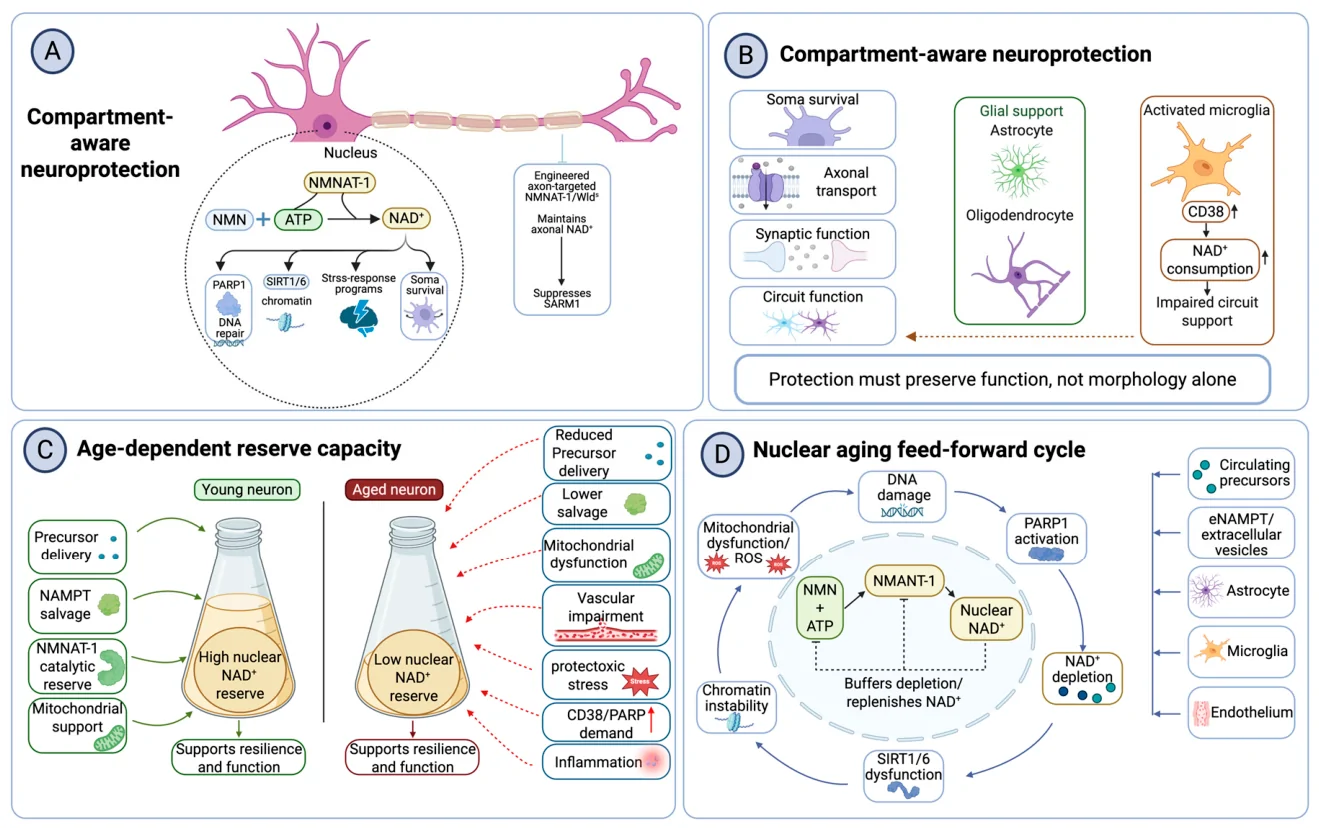
Compartment-specific functions of NMNAT-1 in neuroprotection and neural aging. (**A**) Endogenous NMNAT-1 is predominantly localized to the nucleus, where it catalyzes the conversion of NMN and ATP to NAD^+^ and supports PARP1-dependent DNA repair, SIRT1/6-mediated chromatin regulation, stress-response programs, and neuronal survival. Physiological axonal NAD^+^ maintenance is primarily mediated by NMNAT-2. Following axotomy, loss of axonal NMNAT-2 increases the NMN:NAD^+^ ratio, activates SARM1, promotes NAD^+^ cleavage, and initiates axonal fragmentation. Engineered axon-targeted NMNAT-1 or WldS can compensate for NMNAT-2 depletion and restrain SARM1-dependent degeneration. (**B**) Effective neuroprotection requires the preservation of the complete neural unit, including neuronal soma survival, axonal transport, synaptic transmission, circuit function, and metabolic support from astrocytes and oligodendrocytes. Activated microglia can increase CD38-dependent NAD^+^ consumption and impair circuit support. (**C**) Young neurons maintain a relatively high nuclear NAD^+^ reserve through adequate precursor delivery, NAMPT-mediated salvage, NMNAT-1 catalytic capacity, and mitochondrial support. Aging reduces this reserve through impaired precursor supply and salvage, mitochondrial and vascular dysfunction, proteotoxic stress, inflammation, and increased CD38/PARP demand. NMNAT-1 abundance may remain unchanged even when its effective catalytic reserve becomes substrate- or demand-limited. (**D**) Age-associated DNA damage activates PARP1 and depletes nuclear NAD^+^, leading to SIRT1/6 dysfunction, chromatin instability, mitochondrial impairment, oxidative stress, and further DNA damage. NMNAT-1 buffers this cycle by replenishing nuclear NAD^+^ when sufficient precursors and ATP are available. Circulating precursors, extracellular vesicle-associated eNAMPT, astrocytes, microglia, and endothelial cells additionally influence nuclear NAD^+^ homeostasis. Blue arrows indicate physiological activation, red arrows indicate injury- or aging-related processes, inhibitory bars indicate suppression, and dashed orange arrows indicate NAD^+^ buffering. Created in BioRender. You Sun. (2026) [https://BioRender.com/jjs37z1] (accessed on 18 August 2026).

Glia influence the success of an NMNAT-1 intervention. Schwann cells and oligodendrocytes supply metabolic support and clear degenerating myelin; astrocytes buffer neurotransmitters and provide substrates; microglia remove debris but can also drive chronic NAD^+^ consumption through CD38-rich inflammatory states [9,48]. Preserving an axon while leaving a toxic glial environment unchanged may only postpone degeneration. Conversely, glial NAD^+^ restoration could improve neuronal survival without directly altering neuronal NMNAT-1. Conditional and co-culture experiments are needed to assign these contributions. The compartment-specific protective actions of endogenous and engineered NMNAT-1, together with the need to preserve somatic, axonal, synaptic, and glial function, are summarized in Figure 2A,B.

### 4.7. NAD^+^-Dependent Mechanisms of NMNAT-1 in Neural Aging

During aging, NAD^+^ availability declines in many, although not all, neural tissues and experimental settings. Reduced NAMPT-mediated salvage, increased CD38 activity, persistent PARP activation, mitochondrial dysfunction, chronic inflammation, and altered precursor availability can all reduce the nuclear NAD^+^ pool [9,47,48,49]. These mechanisms do not establish that loss of NMNAT-1 is the universal cause of age-related NAD^+^ decline [1,5]. Rather, nuclear NAD^+^ insufficiency can emerge despite unchanged NMNAT-1 abundance when upstream NMN delivery falls or consumption rises. This distinction is therapeutically important: increasing NMNAT-1 activity requires adequate substrate, whereas precursor supplementation requires sufficient terminal NMNAT-1 catalytic reserve [34,37].

As this metabolic reserve narrows, NMNAT-1-dependent NAD^+^ production becomes increasingly important for genome stability and epigenetic maintenance. Age-associated DNA damage activates PARP enzymes and consumes nuclear NAD^+^; the resulting loss of sirtuin activity can impair chromatin regulation, mitochondrial maintenance, and antioxidant adaptation, thereby creating a feed-forward cycle of oxidative stress and further DNA damage [45,70,72]. NMNAT-1 is positioned to buffer this cycle by replenishing NAD^+^ near chromatin-associated consumers, although the outcome depends on local recruitment and demand rather than on total tissue NAD^+^ alone. In aged neurons, the relevant endpoint is therefore preserved repair capacity and transcriptional precision, not merely a higher steady-state metabolite concentration.

Nuclear NAD^+^ supply also depends on systemic and cell-specific precursor networks. Adipose-derived extracellular NAMPT regulates hypothalamic NAD^+^ and function, extracellular vesicle-associated NAMPT delays selected aging phenotypes in mice, and loss of NAMPT in adult neural stem cells or hippocampal CA1 neurons produces age-related functional defects that can be improved by NMN [50,51,52,53]. These observations place NMNAT-1 at the terminal nuclear step of an inter-organ salvage network rather than as an isolated aging enzyme, consistent with the broader NAD World framework [54]. The ability of NMNAT-1 to protect an aging neural cell therefore reflects both its intrinsic catalytic reserve and the continued delivery of usable precursors.

Age-related inflammation further increases NAD^+^ demand. Senescent-cell signaling can expand CD38-positive immune populations, while activated microglia, astrocytes, endothelial cells, and progenitors adopt metabolic states that alter precursor availability and NAD^+^ consumption [47,48]. By maintaining nuclear NAD^+^, NMNAT-1 may support sirtuin-mediated inflammatory restraint, genome maintenance, and neurovascular adaptation, but the response is likely cell-type specific. NAD^+^ augmentation improves vascular dysfunction, oxidative stress, neurovascular coupling, cognition, and mitophagy in several aged animal models [55,56,57,58], and sirtuin pathways provide a plausible link to synaptic and mitochondrial resilience [4,95,96,97]. These findings support an NAD^+^-dependent aging mechanism, but they do not show that NMNAT-1 is the sole mediator because systemic precursors can be converted by other competent isoforms and can act through vascular, immune, and peripheral tissues [8,34]. The age-dependent contraction of nuclear NAD^+^ reserve and the associated feed-forward cycle of neuronal dysfunction are illustrated in Figure 2C,D.

## 5. Putative NAD^+^-Independent Functions and Disease-Specific Evidence for NMNAT-1

### 5.1. Evidence for NAD^+^-Independent Protection by NMNAT-1

Evidence that mammalian NMNAT-1 can protect neural cells independently of net NAD^+^ synthesis is suggestive but less definitive than the evidence for its catalytic function. The conceptual foundation came from Drosophila, where catalytically impaired Nmnat variants preserved neural integrity under conditions of markedly reduced NAD^+^ synthesis and stress-induced Nmnat associated with Hsp70-containing aggregates and displayed chaperone-like activity [21,22]. These ortholog studies do not establish an identical function for endogenous human NMNAT-1, but they motivated direct biochemical and disease-model tests of NMNAT-1-mediated protein quality control.

For NMNAT-1, the appropriate conclusion is therefore that some protection may be partly independent of measurable catalytic output, rather than that enzymatic function is irrelevant. Residual activity can be biologically meaningful within nuclear microdomains, catalytically inactive variants may retain substrate binding, and bulk NAD^+^ measurements can miss transient local changes [92,93,98]. Conversely, mammalian NMNAT-1 can improve neuronal or behavioral function in tauopathy models even when phospho-tau burden does not consistently decrease [99,100]. A genuine NAD^+^-independent mechanism must therefore be demonstrated with matched protein abundance, folding and localization, compartment-specific flux assays, and reciprocal separation-of-function variants.

### 5.2. Chaperone-like Activity and Client Proteins of NMNAT-1

Biochemical work provides the most direct bridge between NMNAT-1 and proteostasis. NMNAT-1 can bind phosphorylated tau through a surface that overlaps the NAD^+^ substrate-binding region and can suppress amyloid assembly; mammalian assays that included NMNAT-1 likewise showed chaperoning of pathological amyloid clients in vitro [38,39]. In Drosophila, the orthologous Nmnat protein suppresses tau-induced neurodegeneration and promotes clearance of hyperphosphorylated tau oligomers [101]. Because client binding and catalysis use overlapping structural surfaces, substrates, products, and misfolded proteins may compete or allosterically influence one another, creating a direct mechanistic link—and a possible trade-off—between NAD^+^ synthesis and protein quality control.

NMNAT-1 should therefore be viewed as a candidate component of a broader stress-response network rather than as an isolated holdase. Ortholog studies show stress-dependent induction through heat-shock and hypoxia signaling, alternative-splicing-dependent relocalization, and preservation of vulnerable synaptic proteins [62,102,103,104]. For mammalian NMNAT-1, these results generate specific predictions: proteotoxic stress should alter NMNAT-1 abundance, localization, or client occupancy, and those changes should correlate with nuclear aggregate handling even after local NAD^+^ flux is controlled. Until such tests are completed, ortholog evidence should be presented as mechanistic support rather than direct proof of endogenous human NMNAT-1 function.

Disease models nevertheless provide evidence that NMNAT-1 can oppose proteotoxic stress. NMNAT activity reduced mutant huntingtin aggregate toxicity in Drosophila, while mammalian NMNAT-1 improved early behavioral or neuronal function in tauopathy models despite inconsistent effects on phospho-tau abundance [99,100,105]. Human NMNAT-1 expression also promoted autophagic amyloid-plaque clearance in a Drosophila Alzheimer’s model [88]. Together, these observations support a protective role for NMNAT-1 against toxic protein species, but they do not yet establish a universal client-binding mechanism or prove that endogenous NMNAT-1 is limiting in human neurodegenerative disease.

### 5.3. NMNAT-1 in Proteostasis and Stress-Response Integration

Chaperone-like binding by NMNAT-1 could prevent oligomer nucleation, maintain clients in a refolding-competent state, direct them toward degradation, or stabilize vulnerable nuclear and synaptic proteins. Autophagy is implicated in NMNAT-associated protection against hypoxic dendrite degeneration, and autophagic clearance appears in amyloid models [88,89]. An NMNAT-1-centered model therefore couples transient client binding to proteasomal or lysosomal disposal: NMNAT-1 limits formation of toxic assemblies, while ubiquitin-dependent and autophagic pathways complete their removal. This sequence should be tested by measuring flux and cargo clearance rather than relying on static LC3, p62, or aggregate counts.

This model also defines important limits for NMNAT-1. A protein retained in the nucleus cannot directly buffer every cytoplasmic or axonal aggregate, so protection may require stress-induced redistribution, indirect transcriptional effects, or experimentally targeted expression. Excessive client binding could also divert NMNAT-1 from NAD^+^ synthesis, whereas high substrate or product concentrations could alter client occupancy at the shared binding surface [38,39]. Quantitative studies should therefore measure NMNAT-1 catalytic flux, localization, oligomerization, and client occupancy simultaneously rather than assigning a phenotype to either catalysis or chaperoning from a single endpoint.

### 5.4. Criteria for a Genuine NAD^+^-Independent Function of NMNAT-1

**Figure 3:**
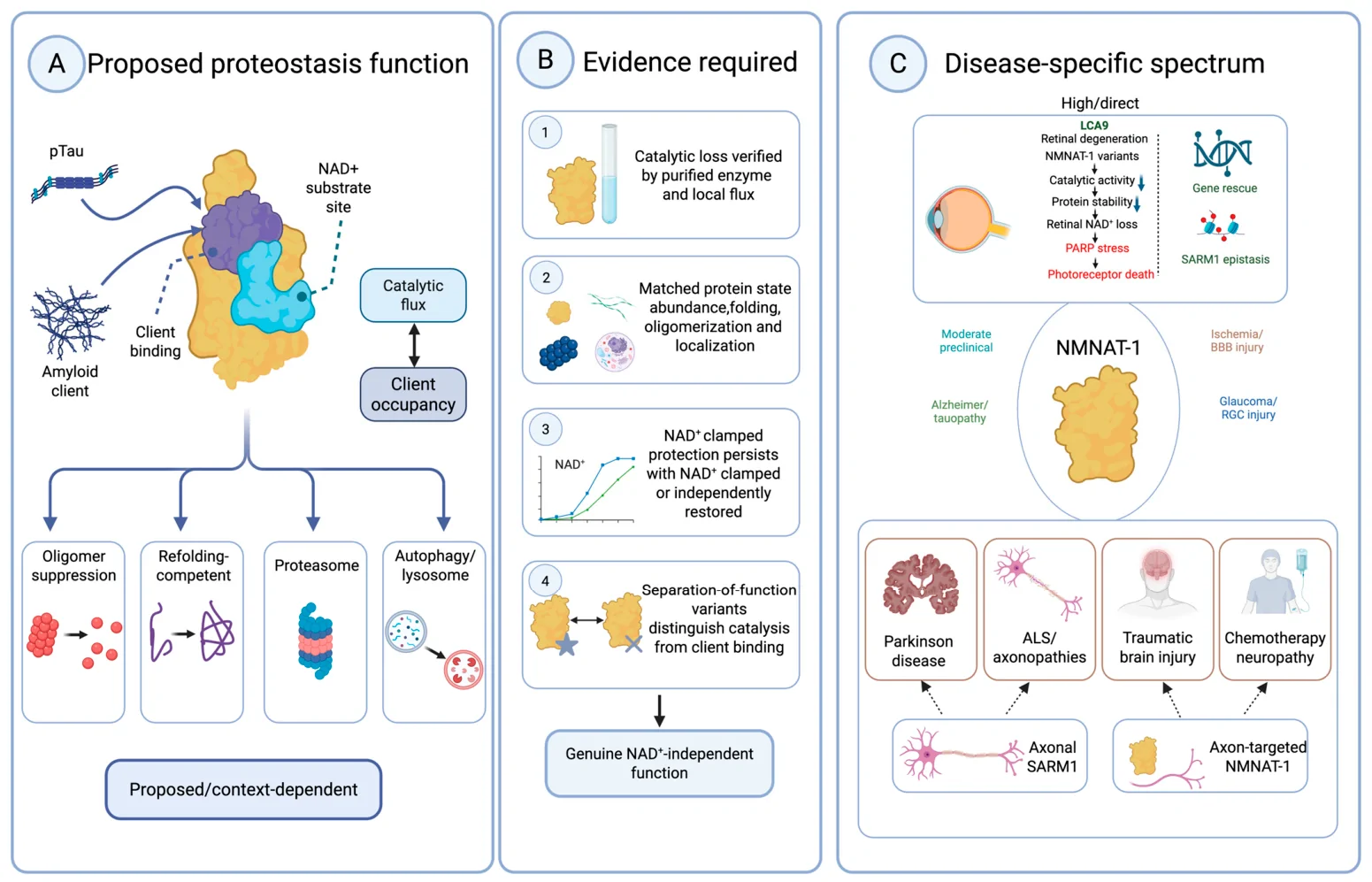
Proposed NAD^+^-independent functions and disease-specific roles of NMNAT-1. (**A**) NMNAT-1 has been proposed to exert chaperone-like or proteostasis-supporting effects through direct interactions with phosphorylated tau and other amyloidogenic clients. Client binding may suppress oligomer formation, maintain proteins in a refolding-competent state, or promote their transfer to proteasomal and autophagy–lysosome degradation pathways. Because the proposed client-binding surface overlaps with the NAD^+^ substrate-binding region, client occupancy and catalytic flux may influence one another. These proteostasis functions remain proposed and context dependent. (**B**) Four criteria are required to demonstrate a genuine NAD^+^-independent function. Catalytic loss must first be verified using purified enzyme and compartment-specific flux measurements. Mutant and wild-type proteins must be matched for abundance, folding, oligomerization, and localization. Protection must persist when NAD^+^ is experimentally clamped or restored independently. Finally, reciprocal separation-of-function variants are required to distinguish catalytic activity from client binding. (**C**) The strength of the NMNAT-1–disease relationship varies across disorders. LCA9 and inherited retinal degeneration provide the most direct evidence through pathogenic NMNAT-1 variants, impaired catalytic activity or protein stability, retinal NAD^+^ loss, PARP stress, photoreceptor death, gene rescue, and SARM1 epistasis. Alzheimer’s disease and tauopathy, cerebral ischemia and blood–brain barrier injury, and glaucoma or retinal ganglion cell injury have moderate preclinical support. Associations with Parkinson’s disease, amyotrophic lateral sclerosis and other axonopathies, traumatic brain injury, and chemotherapy-induced neuropathy remain indirect or emerging and are frequently mediated through axonal SARM1 or experimentally axon-targeted NMNAT-1 rather than endogenous nuclear NMNAT-1. Created in BioRender. You Sun. (2026) [https://BioRender.com/jjs37z1] (accessed on 18 August 2026).

Four criteria would substantially strengthen claims of a true NAD^+^-independent NMNAT-1 function. First, catalytic loss should be verified using purified NMNAT-1 and compartment-specific cellular flux assays, not total NAD^+^ alone. Second, mutant and wild-type NMNAT-1 should be matched for abundance, folding, oligomerization, and localization. Third, protection should persist when NAD^+^ is clamped or restored independently, demonstrating that the phenotype is not caused by an undetected local NAD^+^ change. Fourth, a separable client-binding or chaperone-defective NMNAT-1 variant should abolish protection while preserving catalysis. Reciprocal separation-of-function alleles would provide the most decisive test [92,93]. The proposed proteostasis-related functions of NMNAT-1 and the experimental criteria required to establish genuine NAD^+^ independence are summarized in Figure 3A,B.

### 5.5. NMNAT-1 Reserve Capacity in Neural Aging

Building on the age-dependent framework shown in Figure 2C,D, an NMNAT-1-centered reserve-capacity model proposes that aging narrows the margin between nuclear NAD^+^ supply and episodic demand. A young neuron possesses sufficient precursor delivery, NMNAT-1 activity, and mitochondrial support to absorb bursts of PARP or sirtuin consumption. With age, higher CD38/PARP demand, lower salvage, vascular impairment, and proteotoxic stress reduce this reserve [9,48]. A partial NMNAT-1 defect that is tolerated early may then become pathogenic, whereas a modest increase in nuclear synthesis or a targeted decrease in consumption may restore resilience.

The human relevance of this model remains incompletely resolved. Most mechanistic evidence linking NAD^+^ restoration to neural rejuvenation comes from short-lived organisms, genetically tractable systems, or mice treated before advanced pathology [8,106]. Human aging adds decades of exposure, mixed pathologies, medication effects, and wide variation in diet, exercise, inflammation, and renal or hepatic clearance. Blood NAD^+^ metabolites are sensitive to sampling and processing, while post-mortem brain tissue cannot reconstruct dynamic nuclear flux. A cross-sectional association between low NAD^+^ and cognitive impairment therefore cannot establish whether insufficient NMNAT-1-dependent supply is causal, compensatory, or simply a marker of cell loss.

Human trials of NR and NMN demonstrate that systemic NAD^+^ metabolism is modifiable, but they do not yet show that endogenous NMNAT-1 activity is restored in the aging brain [107,108]. A clinically useful NMNAT-1 biomarker could combine a nuclear NAD^+^-responsive transcriptional signature, PARylation or histone-acetylation markers, and a genotype-informed measure of catalytic reserve. In the retina, optical imaging and electroretinography offer relatively direct functional readouts; in the brain, magnetic resonance spectroscopy, fluid biomarkers, and neuronal extracellular vesicles remain less compartment specific.

The appropriate translational claim is therefore modest but actionable: NMNAT-1 is a strategically placed nuclear node within the NAD^+^ network, but whether it is the limiting node must be demonstrated for each aging context. Studies enriched for an NMNAT-1 variant, low precursor salvage, high nuclear NAD^+^ consumption, or a pathway-defined disorder may be more informative than trials enrolling heterogeneous older adults solely by chronological age. Healthspan, cognitive resilience, and disease-specific function should also be distinguished from lifespan extension, which has not been consistently achieved by NAD^+^-raising interventions [109]. These mechanistic functions of NMNAT-1, together with their compartmental contexts, catalytic requirements, recommended readouts, and major interpretive limitations, are summarized in Appendix A, Table A1.

### 5.6. NMNAT-1 in Neurological and Retinal Disorders

#### 5.6.1. Inherited NMNAT-1-Associated Retinal Degeneration

The strongest human evidence connecting NMNAT-1 to neural survival comes from inherited retinal disease. Initial studies published in 2012 identified biallelic NMNAT-1 variants in Leber congenital amaurosis, usually with profound early visual loss, severe macular involvement, and optic or retinal atrophy [23,24]. Two additional contemporaneous cohorts independently confirmed the association and expanded the allelic spectrum [110,111]. Collectively, these discoveries established LCA9 as a disorder of NAD^+^ biosynthesis and shifted NMNAT-1 from an engineered axon-protection factor to an essential endogenous neural enzyme.

Mouse models reproduce major features of the human disease, including early photoreceptor degeneration and central retinal vulnerability [112]. LCA-associated variants can impair catalytic activity, stability, or both, and genotype alone does not always predict residual biochemical capacity [37]. Retinal progenitor studies show that NMNAT-1 supports survival partly through regulation of pro-apoptotic gene expression and histone acetylation [113]. Strikingly, SARM1 depletion rescues photoreceptor death and retinal degeneration in an NMNAT-1-deficient setting, linking nuclear synthetic failure to a pro-degenerative NADase pathway [114]. Mutant retina also exhibits a selective NAD^+^ decrease with increased poly(ADP-ribose), implicating excess nuclear consumption or stress signaling [115].

Developmental timing and cell identity are critical. Conditional models demonstrate requirements for NMNAT-1 during retinal development and early neuronal survival [42]. A phenotype that begins during lineage specification or photoreceptor maturation cannot necessarily be reversed by adult NAD^+^ supplementation. Conversely, gene therapy preserves retinal structure and function in mouse models, and photoreceptor-directed NMNAT-1 expression can be sufficient for rescue [73,116]. These findings support cell-autonomous need in photoreceptors while leaving room for contributions from retinal pigment epithelium, Müller glia, and inner retinal neurons.

The phenotypic spectrum is broader than classic LCA9. NMNAT-1 variants can cause cone or cone–rod dystrophy, and an E257K allele produces a milder retinal phenotype in experimental models [117,118]. An Alu-mediated duplication causes SHILCA syndrome, which includes spondyloepiphyseal dysplasia, hearing loss, intellectual disability, and retinal degeneration, demonstrating that some alleles disrupt multiple tissues [60]. Clinical reviews emphasize marked allelic and phenotypic heterogeneity. Human induced pluripotent stem cell work further indicates that NMNAT-1 is required for differentiation toward a retinal lineage [74]. Taken together, these data favor a threshold model: residual activity, protein stability, developmental expression, modifier genes, and retinal stress determine which cells cross the threshold for disease.

Why is the retina so vulnerable despite ubiquitous NMNAT-1 expression? No single explanation is sufficient. Photoreceptors have exceptional ATP and NAD(H) turnover, high oxygen exposure, light-driven oxidative stress, daily outer-segment renewal, and extensive transcriptional specialization [42,112]. The macula may face additional structural and metabolic constraints. NMNAT-1 loss can therefore converge on NAD^+^ shortage, PARP stress, epigenetic dysregulation, developmental failure, and SARM1 activation. This convergence also explains why precursor-only therapy may be inadequate when terminal catalytic capacity is severely reduced.

#### 5.6.2. Alzheimer’s Disease and Other Proteinopathies

Direct evidence for NMNAT-1 in Alzheimer’s disease is promising but substantially weaker than in LCA9. In a triple-transgenic Alzheimer’s model, NMNAT-1 reduced mitochondrial oxidative stress and caspase-3 signaling [119]. Earlier NAD^+^ precursor work showed improved mitochondrial function or cognition in Alzheimer’s-relevant mice, while Drosophila and tauopathy studies described in Section 5 support proteostasis-related mechanisms [101,120]. These results provide convergent plausibility but differ in species, construct, compartment, and pathological endpoint.

An important negative or mixed result is that NMNAT-1 can preserve neuronal function without consistently reducing phospho-tau pathology [100]. Functional rescue may therefore arise from improved energy, synaptic resilience, or handling of toxic oligomeric species not captured by bulk phospho-tau measures. Alternatively, behavioral effects may be transient or circuit specific. Future work should use endogenous NMNAT-1 manipulation in human induced neurons and brain organoids, quantify soluble tau conformers, and determine whether protection persists after expression is normalized to physiological levels.

For α-synuclein and Parkinson’s disease, NAD^+^ augmentation rescues mitochondrial and neuronal defects in patient-derived cells and fly models, but NMNAT-1-specific evidence is limited [121]. Wld^S^, not unmodified NMNAT-1, protected dopaminergic neurites from MPP^+^ toxicity in one study, underscoring the importance of the Wld^S^ fusion context [122]. Because axon degeneration can precede neuronal loss in Parkinson’s disease, SARM1 or axonal NAD^+^ may be more direct targets than nuclear NMNAT-1 [123]. The relevant conclusion is not that NMNAT-1 is ineffective, but that localization and disease trigger determine whether it reaches the vulnerable process.

#### 5.6.3. Amyotrophic Lateral Sclerosis and Axonopathies

In amyotrophic lateral sclerosis and related axonopathies, NMNAT-1-specific evidence is indirect and model dependent. Human genetics has implicated hyperactive SARM1 variants in subsets of motor-nerve disease, and Sarm1 deletion suppresses TDP-43-linked motor-neuron degeneration and cortical-spine loss but does not rescue the SOD1G93A model [124,125,126,127]. These findings define a boundary for NMNAT-1 translation: axon-targeted NMNAT-1 could oppose SARM1-mediated NAD^+^ collapse in selected disorders, but nuclear NMNAT-1 augmentation should not be assumed to benefit every ALS mechanism.

The therapeutic rationale for NMNAT-1 is strongest when pathology includes early loss of axonal NMNAT activity, an increased NMN/NAD^+^ ratio, measurable SARM1 activation, or a hyperactive SARM1 genotype. Biomarkers such as cyclic ADP-ribose derivatives, axonal NAD^+^ metabolites, and neurofilament release could identify such cases [6,29]. Nuclear NMNAT-1 may separately improve genome maintenance or proteostasis in motor neurons, but this mechanism must be distinguished experimentally from the engineered use of axon-targeted NMNAT-1 to suppress SARM1.

#### 5.6.4. Cerebral Ischemia, Excitotoxicity, and Blood–Brain Barrier Injury

NMNAT-1 overexpression protects cultured neural cells and mouse brains against ischemic injury with evidence of AMPK activation [90]. In aged rats subjected to transient cerebral ischemia–reperfusion, NMNAT-1-associated protection involved autophagy [128]. A further study implicated an NMNAT-1–NAD^+^–SIRT1 pathway in preservation of the blood–brain barrier after cerebral ischemia [129]. These studies support multiple compartments: neuronal energy stress, autophagic quality control, and vascular integrity.

Interpretation should nevertheless consider construct expression, treatment timing, infarct heterogeneity, and whether catalytic dependence was tested. Ischemia generates abrupt ATP loss, acidosis, excitotoxicity, oxidative DNA damage, inflammation, and vascular leakage. NMNAT-1 is unlikely to control all components equally. A combined intervention that preserves nuclear repair, prevents SARM1-dependent axon loss, and restores perfusion may be more robust than a single NAD^+^-raising maneuver.

In the developing central nervous system, NMNAT-1 protects against acute excitotoxic–necrotic degeneration [130]. Developmental tissue differs from the aged brain in receptor expression, metabolic flexibility, and cell-death programs, so this evidence should not be directly generalized to late-life stroke. It does, however, reinforce the broader principle that sufficient NAD^+^ synthesis can shift the threshold for excitotoxic death.

#### 5.6.5. Retinal Ganglion Cell Injury, Glaucoma, Trauma, and Peripheral Neuropathy

Cytoplasmic NMNAT-1 overexpression protects retinal ganglion cell somata and axons in glaucomatous and ischemic injury [131]. Nicotinamide prevents glaucoma in aged mice by reducing mitochondrial vulnerability, and systemic NR is protective in two retinal ganglion cell damage models [132,133]. These interventions act at different pathway points: NMNAT-1 supplies terminal catalytic activity, precursors increase substrate availability, and their efficacy depends on residual NMNAT function.

Traumatic brain injury and chemotherapy neuropathy clarify the downstream boundary of NMNAT-1-mediated protection. Sarm1 deletion reduces traumatic axonal injury, demyelination, white-matter atrophy, and functional deficits, and it prevents vincristine-induced neuropathy across insults with distinct upstream mechanisms [134,135,136,137]. For NMNAT-1, these findings imply that protection requires sufficient activity in the threatened axonal compartment; endogenous nuclear NMNAT-1 is unlikely to substitute automatically. Axon-targeted NMNAT-1 may be useful in defined settings, but direct SARM1 inhibition is often the more proximal strategy once the degeneration program is activated [87,138].

#### 5.6.6. Glioma and Glioblastoma

Although this review focuses on aging-related neuroprotection, glioma and glioblastoma are relevant because malignant glial cells can co-opt the same nuclear NAD^+^ machinery discussed above. Available evidence should be interpreted separately from neuroprotection. In Drosophila glial neoplasia and human glioma cell models, NMNAT activity supported tumor growth by increasing tolerance to DNA damage and by shifting p53 toward NAD^+^-dependent PARylation and deacetylation states that reduce apoptosis [139]. Additional human-tissue and model data link elevated nuclear NMNAT-1 with nuclear atypia, disrupted lamin A/C distribution, and glioma severity [140]. These findings indicate tumor-supporting dependence, not protection of normal neurons.

A distinct therapeutic concept exploits, rather than inhibits, tumor NMNAT-1 activity. Gliocidin is a nicotinamide-mimetic prodrug whose active metabolite, gliocidin-adenine dinucleotide, is generated through NAD^+^ salvage enzymes, including NMNAT-1; the metabolite blocks IMPDH2-dependent guanine nucleotide synthesis and extends survival in orthotopic glioblastoma models [141]. This creates a carefully bounded translational message: tumor NMNAT-1 may support malignant survival in one context and enable prodrug bioactivation in another. Neither strategy is clinically validated, and both require tumor-selective exposure, biomarker confirmation, resistance monitoring, and safety surveillance.

#### 5.6.7. Evidence Triangulation and Model Limitations

No single disease model captures aging, human genetics, circuit complexity, and treatment exposure. Drosophila excels at genetic dissection and proteostasis studies but has one Nmnat gene and different tissue organization. Rodent overexpression models reveal sufficiency but may produce nonphysiological localization and abundance. Conditional knockout models reveal necessity but can mix developmental and maintenance effects. Human induced pluripotent stem-cell and retinal organoid models can reveal developmental requirements for NMNAT-1 [74], while invertebrate and rodent models remain valuable for dissecting its catalytic, chaperone-like, and neuroprotective functions [142]. Findings from each system should be interpreted as model-specific rather than as complete representations of the mature neurovascular environment.

The most reliable conclusions arise when methods converge. In LCA9, independent human genetic cohorts, biochemical characterization, retinal development models, SARM1 epistasis, and gene rescue point to a causal pathway [23,114]. In sporadic Alzheimer’s or Parkinson’s disease, evidence is distributed across precursor studies, engineered expression, and invertebrate proteinopathy models; the causal position of endogenous NMNAT-1 remains less certain [121,122]. These differences should be visible in review language and in priorities for translation.

Disease stage can also reverse apparent efficacy. Before fragmentation, preserving NAD^+^ may maintain transport and permit recovery. After axonal disintegration or loss of retinal progenitors, metabolic rescue cannot reconstruct anatomy by itself [113]. Some interventions may preserve morphology but trap damaged, electrically silent processes. Functional electrophysiology, behavior, visual responses, and long-term recovery should accompany histology. Where possible, withdrawal experiments should determine whether protection is durable or requires continuous pathway suppression. Figure 3C places these disease associations on an evidence-strength spectrum, whereas the underlying models, therapeutic implications, and major limitations are compared in greater detail in Appendix A Table A2 and Table A3.

## 6. Therapeutic Targeting of NMNAT-1

### 6.1. NAD^+^-Dependent Therapeutic Strategies

#### 6.1.1. NAD^+^ Precursor Supplementation

NMN and NR bypass different upstream steps and can raise NAD^+^ when the relevant kinases, transport routes, and NMNAT activity are intact [8,34]. NMN improves metabolic disease and age-associated phenotypes in mice [57,143]. It supports brain mitochondrial respiration in an Alzheimer-relevant model [144]. It also benefits neurovascular function in aged mice [56,145]. NR enhances oxidative metabolism in mice and is orally bioavailable in humans [146,147]. Repeated dosing raises blood NAD^+^ in healthy middle-aged and older adults and is generally well tolerated in short-duration studies [107].

Clinical neurological evidence is emerging rather than definitive. In Parkinson’s disease, the phase I NADPARK trial showed target engagement and biological responses, and the NR-SAFE trial supported tolerability of high-dose NR [148,149]. A randomized trial in older adults with mild cognitive impairment demonstrated NAD^+^-related biomarker effects but did not establish a broadly effective disease-modifying therapy [106]. Early human studies found that oral NMN increased circulating metabolites and was tolerated [108,150]. In prediabetic women, NMN improved muscle insulin sensitivity, while a multicenter dose-ranging trial further supported short-term tolerability [151,152]. These results establish feasibility, not long-term neuroprotection.

Three bottlenecks limit precursor interpretation. First, blood NAD^+^ is not a surrogate for nuclear NAD^+^ in a specific neural cell [8,34]. Second, a severely defective NMNAT-1 allele may block conversion of NMN to NAD^+^ in the nucleus, so additional NMN could accumulate without solving the terminal defect [29,66]. Third, increasing NMN relative to NAD^+^ in a compromised axon could favor SARM1 activation, although systemic dosing, compartmental kinetics, and intact NMNAT-2 complicate this theoretical risk [81,83]. Trials should measure central target engagement, genotype or pathway biomarkers, and functional outcomes over sufficient duration.

#### 6.1.2. Direct NMNAT-1 Augmentation and Gene Therapy

Direct strategies include increasing transcription, stabilizing the protein, enhancing catalytic efficiency, delivering recombinant enzyme, or using gene augmentation. The retinal setting is the most mature because the target tissue is accessible, the disease is monogenic, and photoreceptor-directed NMNAT-1 expression rescues animal models [73,116]. AAV dose, promoter choice, developmental timing, immune response, and expression level will determine safety and durability.

For brain disease, untargeted NMNAT-1 overexpression is less attractive. Nuclear expression may not protect distal axons, whereas cytoplasmic or axonal targeting could alter NAD^+^ distribution and protein interactions [16,19]. Long-term overexpression may support malignant cell survival or disturb PARP/sirtuin balance [36,68]. A rational vector should therefore match the threatened compartment and cell type—for example, photoreceptor nuclear rescue for LCA9 or an axon-targeted construct for a defined axonopathy—and should include expression levels close to the therapeutic minimum.

Small-molecule NMNAT-1 activators remain an underdeveloped area. Screening is complicated by the conserved active site across NMNAT isoforms and by the need to distinguish increased catalysis from altered protein stability or localization [25,153]. An ideal compound would enhance NMNAT-1 reserve capacity only where substrate and demand justify it, preserve physiological feedback, and avoid supporting tumors. Allosteric stabilization of pathogenic hypomorphic variants may be more selective than global activation [37,118].

#### 6.1.3. Reducing NAD^+^ Consumption: SARM1, CD38, and PARP

Direct SARM1 inhibition can protect axons without requiring delivery of NMNAT-1 to distal processes. Small molecules reproduce key features of SARM1 loss and preserve axons in preclinical injury or chemotherapy models [87,154]. Gene therapy expressing dominant-negative SARM1 also blocks pathological axon degeneration in mice [155]. The strategy is mechanistically attractive because it targets a committed destruction program downstream of many insults.

SARM1 inhibition will not correct nuclear DNA repair, developmental transcription, or all forms of neuronal death [81,83]. It may preserve a disconnected or dysfunctional axon if the upstream disease remains active. Combination with causal therapy is therefore essential. In LCA9, for example, SARM1 inhibition could slow degeneration, while NMNAT-1 gene augmentation restores nuclear synthesis; in chemotherapy neuropathy, transient SARM1 inhibition could protect axons while cancer treatment continues.

CD38 inhibition may preserve NAD^+^ in inflammatory aging, while carefully titrated PARP modulation may reduce catastrophic consumption [9,48]. Both carry trade-offs. CD38 participates in immune and calcium signaling; PARP1 is required for repair and transcriptional responses [48,70]. Complete chronic inhibition may be undesirable, particularly in older or immunocompromised patients. Biomarker-guided, partial, or time-limited inhibition is more plausible than indiscriminate suppression.

#### 6.1.4. Combination Strategies and Therapeutic Matching

The pathway suggests four therapeutic matches [34,87]. When precursor supply is limiting but NMNAT activity is intact, NMN or NR may be sufficient. When NMNAT-1 is hypomorphic, gene augmentation or pharmacological stabilization should be central, with precursor support only if the substrate becomes limiting. When SARM1 is activated in axons, a SARM1 inhibitor can directly prevent NAD^+^ destruction. When PARP/CD38 consumption dominates, reducing demand may be more efficient than increasing supply.

**Figure 4:**
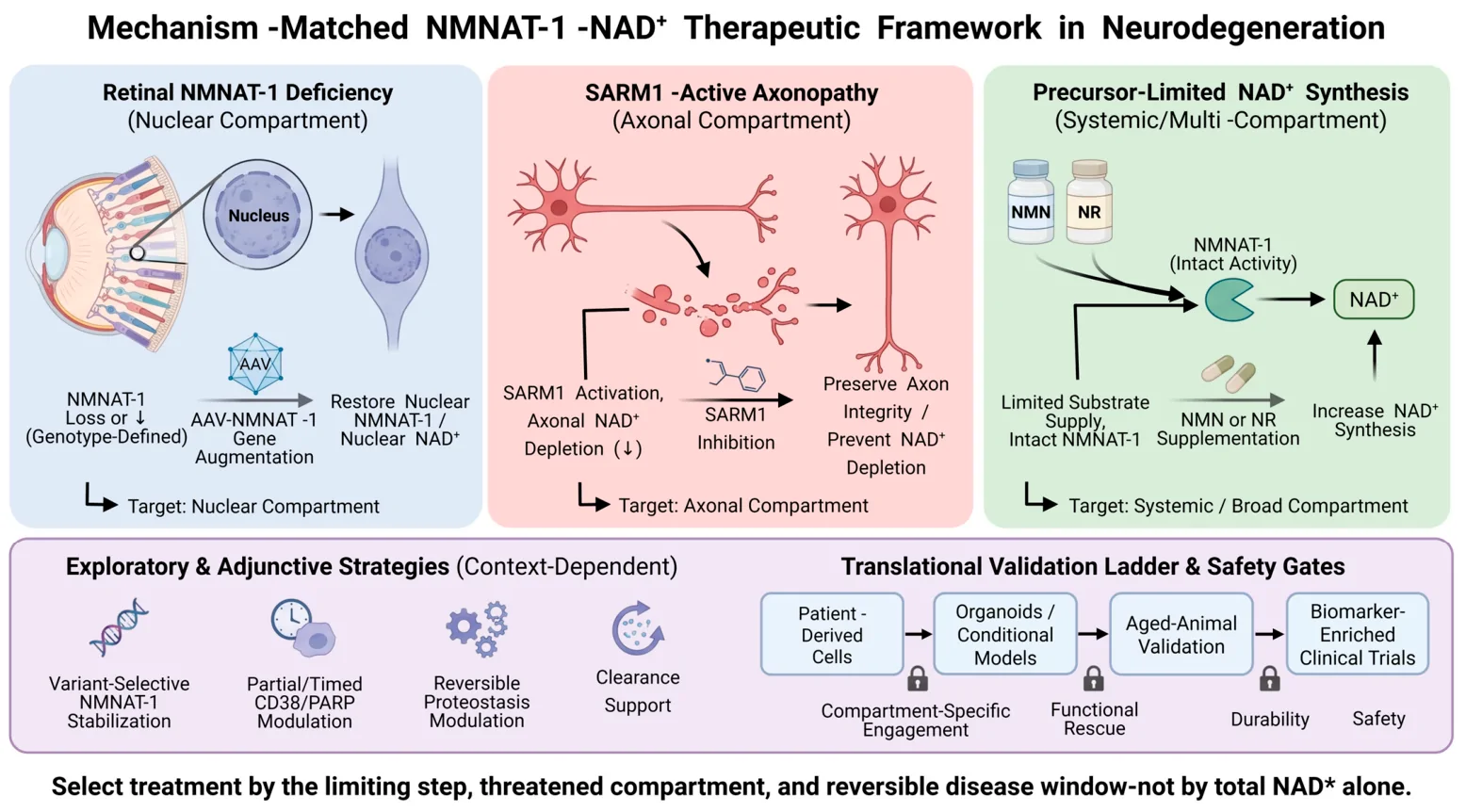
Mechanism-matched NMNAT-1-NAD+ therapeutic framework in neurodegeneration. The revised framework selects treatment according to the limiting pathological step, threatened compartment, and reversible disease window rather than total NAD+ alone. The upper row separates three comparatively better-supported therapeutic scenarios: retinal NMNAT-1 deficiency in the nuclear compartment, addressed by retinal AAV-NMNAT-1 gene augmentation to restore nuclear NMNAT-1/nuclear NAD+; SARM1-active axonopathy in the axonal compartment, addressed by SARM1 inhibition to preserve axon integrity and prevent NAD+ depletion; and precursor-limited NAD+ synthesis in systemic or multi-compartment settings, addressed by NMN/NR supplementation when NMNAT-1 catalytic activity remains intact. The lower band separates context-dependent exploratory or adjunctive strategies, including variant-selective NMNAT-1 stabilization, partial/timed CD38/PARP modulation, reversible proteostasis modulation, and clearance support, from a translational validation ladder. Advancement should proceed from patient-derived cells to organoids/conditional models, aged-animal validation, and biomarker-enriched clinical trials, with compartment-specific target engagement, functional rescue, durability, and safety serving as major gates.

Combination treatment should not merely add every NAD^+^-raising intervention. The relevant variables are flux and compartment. Precursor plus NMNAT-1 augmentation could overproduce NAD^+^ in one compartment while leaving another unchanged. SARM1 inhibition plus axonal NMNAT-1 may be redundant. PARP inhibition plus NMNAT-1 activation could preserve NAD^+^ but impair repair if PARP suppression is excessive. Dose–response matrices, isotope tracing, and local biosensors can identify combinations that restore physiological dynamics rather than maximize a single endpoint. The correspondence between the dominant pathological bottleneck and the appropriate mechanism-matched intervention is summarized in the upper therapeutic framework in Figure 4.

#### 6.1.5. Target Engagement, Patient Selection, and Safety

The largest barrier is target engagement. Because NAD+ pools are compartmentalized and subcellular sensors detect distinct dynamics [15,46], blood or cerebrospinal fluid metabolites should not be assumed to report NAD+ flux in photoreceptor nuclei, cortical neurons, microglia, or distal axons. Candidate biomarkers include compartment-informed metabolite signatures, PARylation, histone acetylation, SARM1 products, neurofilament release, retinal imaging, and electrophysiology [29,115]. No single marker is sufficient; pharmacodynamic panels should link exposure to pathway modulation and tissue function.

Timing is equally important. Developmental retinal deficits require early intervention, whereas an adult axonopathy may offer a reversible window between NMNAT-2 loss and structural fragmentation [28,42]. Chronic neurodegenerative disease may need years of treatment, magnifying safety concerns. NAD^+^ supports repair and normal physiology but can also support malignant cells; SARM1 and CD38 participate in host defense; PARP inhibition can alter genome maintenance [5,70]. Human trials must therefore measure cancer incidence, immune function, liver and metabolic effects, and potential rebound after withdrawal.

Finally, patient selection should reflect the mechanism. An NMNAT-1 genotype, demonstrable SARM1 activation, low NAD^+^ salvage capacity, or a defined inflammatory CD38 signature is more actionable than age alone [29,48]. Precision targeting is likely to produce larger effects with lower exposure than universal NAD^+^ boosting.

#### 6.1.6. A Staged Development Pathway

A practical development program can proceed in four stages. Stage 1 establishes biochemical correction in patient-derived cells using compartment-specific NAD^+^ sensors and isotope tracing. Stage 2 tests cell-type and timing requirements in organoids or conditional animal models. Stage 3 links dose to a translational biomarker—retinal electrophysiology, SARM1 products, PARylation, or a validated nuclear NAD^+^ signature. Stage 4 evaluates functional durability and delayed toxicity in aged animals before human efficacy trials. Skipping the first three stages risks a negative trial that cannot distinguish target failure from delivery failure.

For monogenic retinal disease, genotype-specific information should guide the modality. A null or unstable protein favors gene replacement; a catalytically hypomorphic but stable protein may respond to an allosteric stabilizer; a variant that disrupts localization may require corrected targeting [37,116]. For an acquired axonopathy with intact NMNAT-1, SARM1 inhibition may be more direct than nuclear gene augmentation [81,83]. For systemic age-related decline, precursor supplementation may be justified only after documenting adequate terminal conversion and central target engagement [8,34].

Manufacturing and dosing constraints also shape biological design. AAV expression is long-lasting and difficult to withdraw, favoring conservative promoters and tissue restriction [73,116]. Small-molecule SARM1 inhibitors are reversible but require sustained axonal exposure [87,156]. NR and NMN are easy to administer but generate broad systemic metabolites and variable tissue delivery [8,34]. The safest intervention is not necessarily the weakest; it is the one whose exposure, reversibility, and compartment match the pathological window.

Finally, success criteria should be prespecified. A biochemical increase without functional improvement may be valuable for dose selection but is not clinical efficacy. Conversely, a functional benefit without demonstrated target engagement is difficult to optimize and reproduce. Integrating mechanism, pharmacology, and patient selection from the outset will allow the NMNAT-1–NAD^+^ field to move beyond undifferentiated supplementation toward rational neuroprotection.

### 6.2. NAD^+^-Independent Therapeutic Strategies

#### 6.2.1. Enhancing NMNAT-1 Chaperone-like and Proteostasis Functions

A distinct therapeutic strategy would enhance the chaperone-like or proteostasis-supporting activity of NMNAT-1 without requiring a net increase in NAD^+^ synthesis. Candidate approaches include stabilizing the native NMNAT-1 fold, increasing its availability to toxic phosphorylated or amyloidogenic clients, promoting transfer of bound clients to the proteasome or autophagy–lysosome system, and using tissue-restricted expression of engineered separation-of-function variants [38,39,88]. This approach may be relevant to proteinopathies in which soluble oligomers or impaired aggregate clearance are more proximal than NAD^+^ depletion.

The structural overlap between the catalytic pocket and client-binding surface makes selective modulation difficult [38,153]. A compound that strengthens client binding could inhibit catalysis, whereas a catalytic activator could weaken chaperone-like interactions. Screening should therefore quantify NMNAT-1 enzymatic flux and client handling in parallel, followed by tests of aggregate toxicity, autophagic completion, neuronal function, and subcellular localization. The goal is not maximal binding but a reversible increase in proteostasis capacity that preserves the essential nuclear NAD^+^-synthetic function of NMNAT-1.

#### 6.2.2. Validation and Translational Limits of NAD^+^-Independent Targeting

Translation of an NAD^+^-independent strategy requires evidence that cannot be supplied by total NAD^+^ measurements alone. Target engagement should include NMNAT-1–client interaction, aggregate-state or soluble-oligomer assays, proteasomal or autophagic flux, and preservation of neuronal function while compartment-specific NAD^+^ flux remains unchanged. Catalytic-dead constructs are insufficient unless their folding, localization, stability, oligomerization, and residual activity are matched to wild-type NMNAT-1 [92,93]. Reciprocal variants that selectively disrupt client binding while preserving catalysis are essential for establishing the mechanism.

Safety considerations also differ from those of precursor therapy. Long-lived NMNAT-1 expression could stabilize abnormal proteins, interfere with physiological protein turnover, or support survival of damaged and potentially malignant cells [36,68]. Because this branch remains preclinical, the initial priority should be reversible, cell-type-restricted modulation in human induced neurons, retinal organoids, and aged animal models. Advancement should require durable functional rescue, absence of pathological aggregate redistribution, preserved NAD^+^-dependent repair, and no evidence of proliferative or oncogenic support. Figure 4 integrates the three mechanism-matched therapeutic scenarios with exploratory or adjunctive strategies, a translational validation ladder, target-engagement requirements, functional endpoints, durability, and safety safeguards; a detailed comparison is provided in Appendix A, Table A4.

### 6.3. Translational Barriers and Go/No-Go Criteria

Four barriers should be treated as go/no-go gates rather than general caveats. First, target engagement must be demonstrated in the relevant compartment: photoreceptor nuclei, cortical neurons, microglia, vascular cells, tumor cells, or distal axons. Second, delivery and timing must match the biological window; developmental retinal defects, acute axonopathy, chronic neurodegeneration, and glioblastoma require different exposure profiles. Third, long-term safety and reversibility must be explicit because NAD^+^ supply can support DNA repair and normal physiology but may also sustain damaged or malignant cells. Fourth, patient selection should be mechanism-based, using NMNAT-1 genotype, SARM1 activation, precursor-salvage status, CD38/PARP demand, tumor NMNAT-1 expression, or compartment-informed biomarkers.

A development program should stop or be redesigned when biochemical correction fails, when pathway modulation cannot be linked to functional rescue, when protection preserves morphology without durable function, or when proliferative, immune, genomic-maintenance, hepatic, metabolic, or rebound risks outweigh the expected benefit. These gates are especially important for AAV-based NMNAT-1 augmentation, long-duration SARM1 inhibition, broad NMN/NR supplementation, CD38/PARP modulation, and gliocidin-like tumor strategies.

## 7. Conclusions, Evidence Limits, and Priority Knowledge Gaps

Four priority knowledge gaps define the next stage of the field. Gap 1 is endogenous human NMNAT-1 physiology during aging: studies should determine how NMNAT-1 abundance, localization, catalytic flux, and partner occupancy change across neuronal, glial, vascular, retinal, and peripheral compartments. Gap 2 is rigorous separation of catalytic and client-binding functions: reciprocal separation-of-function variants are needed to test whether chaperone-like protection persists when local NAD^+^ flux is controlled and whether client-binding disruption abolishes protection while catalysis is preserved.

Gap 3 is disease and compartment specificity. Retinal vulnerability, the boundary between endogenous nuclear NMNAT-1 and engineered axonal NMNAT-1/WldS, SARM1 rescue in NMNAT-1-deficient retina, and NMNAT-1 dependence in glioma should be compared with resistant tissues carrying similar pathway perturbations. Gap 4 is translational readiness: candidate therapies require biomarkers for local flux, NMN/NAD^+^ ratio, PARylation, histone acetylation, SARM1 activity, tumor NMNAT-1 expression, durability, reversibility, and long-term safety before efficacy claims are advanced.

More broadly, the field needs standardized reporting. Studies should specify species and isoform, construct localization, expression relative to endogenous protein, catalytic activity, local and total NAD^+^, NADH and NMN, cell type, age, sex, injury stage, tumor context when relevant, and whether protection preserves function rather than morphology alone. These standards would make apparently conflicting studies comparable and accelerate translation.

Strong/direct evidence supports NMNAT-1 as an essential nuclear NAD^+^ synthase and a causal gene in inherited retinal degeneration. Moderate/convergent preclinical evidence supports protection when NMNAT activity is delivered to the appropriate compartment in selected retinal, ischemic, tauopathy, and axon-injury models. Evidence for a general role of endogenous NMNAT-1 in human brain aging or sporadic neurodegeneration remains indirect, heterogeneous, or pathway-adjacent, and NAD^+^ precursor feasibility should not be equated with NMNAT-1-specific neural efficacy.

Putative NAD^+^-independent chaperone-like activity remains preliminary until catalysis, localization, folding, oligomerization, local NAD^+^ flux, and client binding are separated rigorously in mammalian neurons or retinal cells. Likewise, glioma evidence shows that NMNAT-1 can be disease-promoting or therapeutically exploitable in tumor contexts, not that global NMNAT-1 activation is universally beneficial. No human trial has yet established the efficacy of an NMNAT-1-directed therapy. A compartment-aware, evidence-stratified approach therefore transforms NMNAT-1 from a generic NAD^+^-boosting target into a precise, testable node for neuroprotective and disease-specific intervention.

## Data Availability

No new data were created or analyzed in this study. Data sharing is not applicable to this article.

## Abbreviations

The following abbreviations are used in this manuscript:

NAD^+^	Nicotinamide adenine dinucleotide, oxidized form
DNA	Deoxyribonucleic acid
NMNAT-1	Nicotinamide mononucleotide adenylyltransferase 1
NMNAT	Nicotinamide mononucleotide adenylyltransferase
PAR	Poly(ADP-ribose)
ADP	Adenosine diphosphate
SARM1	Sterile alpha and Toll/interleukin-1 receptor motif-containing protein 1
PARP1	Poly(ADP-ribose) polymerase 1
NADH	Nicotinamide adenine dinucleotide, reduced form
PARP/PARPs	Poly(ADP-ribose) polymerase/polymerases
CD38	Cluster of differentiation 38
TIR	Toll/interleukin-1 receptor
ATP	Adenosine triphosphate
NMN	Nicotinamide mononucleotide
NaMN	Nicotinic acid mononucleotide
NMNAT-2	Nicotinamide mononucleotide adenylyltransferase 2
NMNAT-3	Nicotinamide mononucleotide adenylyltransferase 3
WldS	Wallerian degeneration slow protein
UBE4B	Ubiquitination factor E4B
LCA9	Leber congenital amaurosis type 9
SLC25A51	Solute carrier family 25 member 51
NAMPT	Nicotinamide phosphoribosyltransferase
SIRT1	Sirtuin 1
SIRT6	Sirtuin 6
SIRT7	Sirtuin 7
mRNA	Messenger ribonucleic acid
NR	Nicotinamide riboside
rRNA	Ribosomal ribonucleic acid
NAPRT	Nicotinate phosphoribosyltransferase
Sir2	Silent information regulator 2
RNA	Ribonucleic acid
MCART1	Mitochondrial carrier triple repeat 1
PARylation	Poly(ADP-ribosyl)ation
NF-κB	Nuclear factor kappa B
NADase	Nicotinamide adenine dinucleotide glycohydrolase
MAP kinase	Mitogen-activated protein kinase
PGC-1α	Peroxisome proliferator-activated receptor gamma coactivator 1 alpha
NADPH	Nicotinamide adenine dinucleotide phosphate, reduced form
NAD(H)	Combined oxidized and reduced nicotinamide adenine dinucleotide pool
AMPK	Adenosine monophosphate-activated protein kinase
mTOR	Mechanistic target of rapamycin
LC3	Microtubule-associated protein 1 light chain 3
p62	Sequestosome 1/autophagy receptor p62
CA1	Cornu ammonis area 1
eNAMPT	Extracellular nicotinamide phosphoribosyltransferase
Hsp70	Heat shock protein 70
LCA	Leber congenital amaurosis
E257K	Glutamate-to-lysine substitution at amino acid residue 257
SHILCA	Spondyloepiphyseal dysplasia, sensorineural hearing loss, intellectual disability, and Leber congenital amaurosis
MPP^+^	1-Methyl-4-phenylpyridinium ion
TDP-43	TAR DNA-binding protein 43
SOD1G93A	Superoxide dismutase 1 with a glycine-to-alanine substitution at residue 93
ALS	Amyotrophic lateral sclerosis
NADPARK	NAD supplementation in drug-naive Parkinson’s disease trial
NR-SAFE	Safety trial of high-dose nicotinamide riboside in Parkinson’s disease
AAV	Adeno-associated virus
NaAD	Nicotinic acid adenine dinucleotide
iPSC	Induced pluripotent stem cell
ERG	Electroretinography
OCT	Optical coherence tomography
AD	Alzheimer’s disease
pTau	Phosphorylated tau
BBB	Blood–brain barrier
RGC	Retinal ganglion cell
TBI	Traumatic brain injury
GBM	Glioblastoma
GAD	Gliocidin-adenine dinucleotide
IMPDH2	Inosine monophosphate dehydrogenase 2

## Appendix A

**Table A1:** Mechanistic functions of NMNAT-1 and the evidence required to interpret them.

NMNAT-1 Function	Dominant Compartment	Molecular Event	Representative Evidence	Catalytic Dependence	Recommended Readouts	Interpretive Limitation	Refs.
Terminal NAD^+^ synthesis	Nucleus; soluble nucleoplasm, chromatin and nucleolus	ATP-dependent adenylylation of NMN or NaMN generates NAD^+^ or NaAD	Recombinant enzyme and structural studies; pathogenic variants reduce activity and/or stability	Directly catalytic	Isotope-resolved nuclear flux; NAD^+^/NADH; NMN/NAD^+^; ATP; protein abundance and localization	Steady-state total NAD^+^ cannot distinguish higher synthesis from lower consumption	[10,11,25,35,37,153]
Nuclear NAD^+^ microdomain formation	Promoters, DNA lesions and nucleolus	Local coupling of NMNAT-1-derived NAD^+^ to PARP1 and transcriptional machinery	Promoter-directed recruitment, phosphorylation-dependent PARP1 regulation and rRNA-transcription studies	Catalysis is central; recruitment and modification regulate access	Local NAD^+^ sensor; PARylation; DNA-repair kinetics; locus-specific transcription; NMNAT-1 proximity labeling	Bulk metabolite assays can miss transient local flux and locus specificity	[15,16,44,64,65,70,71,72]
Sirtuin and chromatin support	Nuclear chromatin with downstream mitochondrial effects	NAD^+^ supply supports SIRT1/6/7-dependent deacetylation, stress adaptation and transcriptional precision	Nuclear salvage enzymes regulate SIRT1 at promoters; aging studies link NAD^+^ loss to sirtuin dysfunction	NAD^+^ dependent, but not necessarily NMNAT-1 limited in every context	Histone acetylation; sirtuin activity; chromatin accessibility; mitochondrial gene programs; oxidative stress	Systemic NAD^+^ augmentation can act through other isoforms, vascular cells or peripheral tissues	[45,49,50,51,52,53,54,55,56,57,58,61,64,95,96,97]
Engineered axon protection	Cytoplasm and distal axon after retargeting or fusion	Local NAD^+^ preservation and suppression of the NMN/NAD^+^-sensing SARM1 destruction program	WldS, axon-targeted NMNAT-1, protein transduction and transgenic overexpression delay degeneration	Usually requires NMNAT activity; potency strongly depends on localization	Axonal NAD^+^ and NMN/NAD^+^; SARM1 products; fragmentation; conduction; long-term functional recovery	Endogenous nuclear NMNAT-1 is not equivalent to NMNAT-2, WldS or axon-targeted constructs	[17,18,19,20,28,29,33,75,76,77,78,79,80,81,82,83,84,85,86,87,92,93,94]
Retinal development and survival	Photoreceptor and retinal-progenitor nuclei; additional retinal cell types may contribute	Maintains nuclear NAD^+^, epigenetic regulation and survival thresholds; deficiency can engage PARP and SARM1-linked degeneration	Human biallelic variants, biochemical characterization, mouse models, iPSC differentiation, SARM1 epistasis and gene rescue	Strongly linked to residual catalytic capacity and protein stability	Retinal NAD^+^; PARylation; histone acetylation; ERG; OCT; photoreceptor survival; developmental timing	Precursor-only therapy may fail when terminal NMNAT-1 conversion is severely impaired	[23,24,37,42,47,48,60,73,74,110,111,112,113,114,115,116,117,118]
Chaperone-like client binding	Primarily nuclear for endogenous NMNAT-1; other compartments in engineered or ortholog models	Substrate-binding surface engages phosphorylated tau and other amyloidogenic clients and suppresses assembly	Direct biochemical binding and aggregation assays; Drosophila catalytic-impairment and tau studies	Potentially partly independent of net NAD^+^ synthesis; catalytic and client-binding surfaces overlap	Client occupancy; soluble oligomers; aggregation kinetics; catalytic flux; folding, oligomerization and localization controls	Evidence is strongest biochemically and in ortholog models; endogenous human neuronal function remains unproven	[21,22,38,39,92,93,99,100,101,102,103,104,105,142]
Proteostasis and clearance integration	Nucleus, soma and dendrites depending on construct and stress state	Transient holdase-like action may couple toxic clients to proteasomal or autophagy–lysosome disposal	Autophagy contributes to Nmnat-mediated protection in hypoxic dendrite and amyloid models	May include both NAD^+^-dependent autophagy signaling and non-catalytic client handling	Autophagic flux; lysosomal completion; proteasome activity; cargo clearance; neuronal function rather than static LC3/p62 alone	Static aggregate or autophagy-marker counts cannot prove successful clearance	[88,89,101,102,103,104,105,128]
Aging reserve capacity	Neuronal and glial nuclei within systemic precursor and inflammatory networks	Declining supply and rising CD38/PARP demand narrow the margin between NAD^+^ synthesis and episodic consumption	Aging and NAD^+^-augmentation studies support the network; direct human NMNAT-1 limitation is not established	Conceptually NAD^+^ dependent; whether NMNAT-1 is the limiting node is context specific	Compartment-specific flux; PARylation; histone acetylation; inflammatory/CD38 signatures; cognitive, vascular and retinal function	Chronological age or blood NAD^+^ alone is insufficient for patient selection	[5,9,47,48,49,50,51,52,53,54,55,56,57,58,95,96,97,106,107,108,109]
Context-dependent glioma support	Glioma and glioblastoma nuclei	Nuclear NAD^+^ synthesis may support DNA-damage tolerance, p53 PARylation/deacetylation, lamina remodeling and tumor growth	Drosophila glial neoplasia, human glioma cell models, patient expression/survival associations and nuclear-atypia studies	NAD^+^ dependent in available models; enzymatic activity appears important	NMNAT-1 abundance/localization; PARP1/SIRT1-p53 PTMs; apoptosis; lamin A/C distribution; proliferation and survival	Tumor-supporting dependence is not neuroprotection and does not justify untargeted neural activation	[139,140]

**Table A2:** Direct and convergent disease-specific evidence for NMNAT-1 and related NAD^+^/SARM1 pathway interventions.

Disease/Injury	NMNAT-1 Evidence and Model	Likely Compartment/Mechanism	Key Findings	Strength of Linkage	Therapeutic Implication	Major Caveat	Refs.
LCA9 and related inherited retinal degeneration	Independent human genetic cohorts; mutant biochemistry; retinal conditional and knock-in models; human iPSC retinal differentiation	Photoreceptor/progenitor nuclear NAD^+^ synthesis; PARP stress, histone regulation and SARM1-linked degeneration	Early severe macular/retinal disease; allele-dependent activity/stability; SARM1 depletion and NMNAT-1 gene therapy rescue models	High: direct human causality with convergent mechanistic rescue	Early, cell-targeted gene augmentation; variant-selective stabilization; possible SARM1 combination	Developmental loss and severe terminal catalytic failure may limit precursor-only rescue	[23,24,37,42,47,48,60,73,74,110,111,112,113,114,115,116,117,118]
Alzheimer’s disease and tauopathy	NMNAT-1 overexpression in tau/AD mouse models; human NMNAT-1 in Drosophila amyloid model; biochemical pTau binding	Nuclear/mitochondrial stress and proteostasis; possible client binding plus NAD^+^-dependent resilience	Improved neuronal or behavioral function and reduced oxidative/caspase stress; phospho-tau burden is not consistently reduced	Moderate preclinical, low direct human causal evidence	Test physiological NMNAT-1 modulation in human induced neurons; focus on soluble oligomers and functional rescue	Construct, species, expression level and pathological endpoint differ; benefit may not reflect endogenous limitation	[38,39,88,99,100,101,119,120]
Cerebral ischemia, excitotoxicity and BBB injury	NMNAT-1 overexpression in cultured neurons, mouse brains and aged-rat ischemia; BBB study implicating NAD^+^–SIRT1	Neuronal energy stress, nuclear repair, autophagy and vascular integrity	Reduced ischemic injury with AMPK/autophagy involvement and improved BBB preservation	Moderate preclinical	Combine nuclear metabolic support with perfusion/vascular therapy and, when indicated, SARM1 suppression	Abrupt ATP failure may limit synthesis; construct level, timing and catalytic dependence vary	[90,128,129,130]
Retinal ganglion-cell injury and glaucoma	Cytoplasmic NMNAT-1 overexpression plus nicotinamide and NR studies in glaucoma/RGC damage models	RGC soma and axon; mitochondrial vulnerability and terminal precursor conversion	Protection of soma and axons; aged-mouse glaucoma prevention and systemic precursor benefit	Moderate preclinical	Match precursor support to residual NMNAT capacity; consider axonal targeting and accessible retinal functional monitoring	Different interventions act at different pathway steps and cannot be considered mechanistically equivalent	[131,132,133]
Glioma and glioblastoma	Human expression/survival associations; Drosophila and human glioma models; NMNAT-1-dependent gliocidin bioactivation in orthotopic GBM models	Tumor nuclear NAD^+^ demand, p53 PTMs, lamina remodeling and prodrug activation	High NMNAT-1 associates with poorer outcome and nuclear atypia; NMNAT activity supports growth in models; gliocidin requires NMNAT-1-dependent conversion to active metabolite	Moderate for tumor biology; preclinical for gliocidin; not validated clinically	Consider tumor-selective NMNAT-1 inhibition or NMNAT-1-dependent prodrug activation only with target-engagement biomarkers	Opposite therapeutic logic from neuroprotection; risk of supporting malignant survival if NMNAT-1 is broadly augmented	[139,140,141]

**Table A3:** Indirect and pathway-adjacent evidence for NMNAT-1 and related NAD^+^/SARM1 pathway interventions.

Disease/Injury	NMNAT-1 Evidence and Model	Likely Compartment/Mechanism	Key Findings	Strength of Linkage	Therapeutic Implication	Major Caveat	Refs.
Parkinson’s disease/α-synuclein-related degeneration	NR benefits patient-derived and fly models; WldS, but not unmodified NMNAT-1, protected MPP^+^-treated dopaminergic neurites in one study	Mitochondrial NAD^+^ and early axon degeneration may dominate over nuclear NMNAT-1	NAD^+^ augmentation is biologically active, but NMNAT-1-specific neuroprotection remains limited	Indirect/emerging	Prioritize central target engagement and axonal SARM1 biomarkers; distinguish precursor effects from NMNAT-1 effects	WldS cannot be treated as equivalent to endogenous NMNAT-1; localization is decisive	[121,122,123,148,149]
ALS and motor axonopathies	Human hyperactive SARM1 variants in subsets; Sarm1 deletion benefits TDP-43 but not SOD1G93A models	Distal axon SARM1 pathway; separate possible nuclear repair/proteostasis roles	Model-dependent suppression of motor-neuron and cortical-spine degeneration	Indirect and heterogeneous	Enroll pathway-positive cases with SARM1 activity, high NMN/NAD^+^ or relevant genotype; direct SARM1 inhibition may be preferable	Not every ALS mechanism is SARM1 dependent; nuclear NMNAT-1 augmentation should not be generalized	[124,125,126,127]
Traumatic brain injury	Sarm1 deletion reduces traumatic axonal injury, demyelination and white-matter atrophy	Axonal SARM1 execution pathway downstream of diverse upstream damage	Improved structural and functional outcomes after experimental TBI	Strong for SARM1; indirect for NMNAT-1	SARM1 inhibition is more proximal than untargeted nuclear NMNAT-1 augmentation; axon-targeted NMNAT-1 remains a testable alternative	Preserving axons without correcting the primary lesion may delay rather than reverse dysfunction	[134,135]
Chemotherapy-induced peripheral neuropathy	Genetic and pharmacological SARM1 blockade in vincristine, bortezomib and paclitaxel models	Peripheral axon SARM1 pathway; sufficient local NAD^+^ supply is the NMNAT-related boundary	Axon protection across distinct chemotherapy triggers	Strong for SARM1; indirect for NMNAT-1	Reversible SARM1 inhibition during chemotherapy may be more direct than NMNAT-1 gene delivery	Must preserve antitumor efficacy and avoid supporting damaged or dysfunctional axons indefinitely	[87,136,137,138,154,156]

**Table A4:** Therapeutic strategies, best-fit biological defects, validation requirements and translational limits.

Strategy	Best-Fit Defect/Population	Target Compartment and Mechanism	Required Target-Engagement Panel	Functional Endpoint	Principal Advantage	Key Risks/Limitations	Status and Refs.
NMN or NR supplementation	Low precursor supply or salvage with intact terminal NMNAT conversion	Systemic precursor network feeding neural/cellular NAD^+^ pools	Central or cell-specific NAD^+^ flux, NMN/NAD^+^, precursor metabolites and nuclear transcriptional response—not blood NAD^+^ alone	Cognition, neurovascular coupling, mitochondrial function, disease-specific clinical scale	Oral, reversible and broadly deployable	Variable brain delivery; severe NMNAT-1 defects may block conversion; theoretical NMN accumulation/SARM1 risk; efficacy remains uncertain	Human target engagement and safety established; neurological efficacy emerging. [8,34,56,57,58,106,107,108,109,143,144,145,146,147,148,149,150,151,152]
NMNAT-1 gene augmentation	Null/unstable NMNAT-1 alleles, especially genotype-defined retinal disease	Photoreceptor or retinal-progenitor nucleus; restores terminal NAD^+^ synthesis	Vector expression, nuclear localization, retinal NAD^+^, PARylation, histone acetylation, OCT and ERG	Preserved retinal structure, visual response and durable cell survival	Addresses the causal lesion and can be cell targeted	AAV dose, developmental window, immune response, long-lived exposure and difficult reversibility; excessive survival signaling	Strong preclinical retinal rescue. [42,73,116]
Variant-selective NMNAT-1 stabilization or activation	Stable hypomorphic or folding-defective protein with residual catalytic potential	Nuclear NMNAT-1 abundance, folding or catalytic reserve	Purified and cellular catalysis, thermal stability, localization, isoform selectivity and compartment-specific flux	Genotype-specific rescue of retinal/neural function	Potentially more titratable and selective than global gene overexpression	Conserved active site; no mature selective activators; may alter client binding or support malignant survival	Discovery stage. [25,36,37,63,68,118,153]
Axon-targeted NMNAT-1 or WldS-inspired constructs	Defined SARM1-dependent axonopathy before irreversible fragmentation	Distal axon; preserves NAD^+^ and suppresses SARM1 activation	Axonal localization and expression, NMN/NAD^+^, SARM1 products, transport and conduction	Axon integrity plus electrophysiological and behavioral recovery	High local potency when localization matches the threatened compartment	Nonphysiological expression and targeting; endogenous NMNAT-1 is not equivalent; may preserve dysfunctional axons	Preclinical sufficiency. [17,18,19,20,33,75,76,77,78,79,92,93,94]
SARM1 inhibition	Pathway-positive axonopathy, TBI or chemotherapy neuropathy	Axonal NADase destruction program downstream of NMNAT loss/imbalance	SARM1 catalytic products, axonal NAD^+^, neurofilament, morphology and conduction	Preserved function and recovery after withdrawal, not morphology alone	Direct, reversible blockade of a common committed degeneration program	Does not restore nuclear repair or causal disease; may preserve disconnected axons; sustained exposure may be required	Advanced preclinical small molecules and gene therapy. [6,29,81,82,83,84,85,86,87,134,135,136,137,138,154,155,156]
Partial CD38 or PARP demand reduction	Inflammatory aging with CD38 excess or catastrophic PARP-driven NAD^+^ consumption	Immune/glial and nuclear NAD^+^ consumption	CD38 cell-state signature, PARylation, DNA-repair competence, inflammatory markers and compartment-specific NAD^+^	Neurovascular, cognitive, inflammatory and genomic-stability endpoints	Targets pathological demand when increasing supply is inefficient	CD38 supports immune/calcium signaling; PARP1 supports repair/transcription; chronic complete inhibition is undesirable	Mechanistically plausible, biomarker-guided use needed. [9,47,48,70,71,72]
Proteostasis-directed NMNAT-1 modulation	Proteinopathy dominated by toxic soluble clients or impaired clearance	NMNAT-1 client-binding surface with proteasome/autophagy coupling	NMNAT-1–client interaction, soluble oligomers, aggregate kinetics, proteasomal/autophagic flux and unchanged local NAD^+^ flux	Durable neuronal/synaptic rescue without pathological aggregate redistribution	Could target toxic protein species without requiring global NAD^+^ elevation	Catalytic and client-binding surfaces overlap; risk of inhibiting NAD^+^ synthesis or stabilizing abnormal proteins; mechanism not validated clinically	Early preclinical/biochemical. [21,22,38,39,88,89,92,93,99,100,101,102,103,104,105]
NMNAT-1-dependent gliocidin bioactivation	NMNAT-1-expressing glioblastoma with IMPDH2-dependent guanine-synthesis vulnerability	Tumor-cell NAD^+^ salvage converts gliocidin to gliocidin-adenine dinucleotide, which blocks IMPDH2	Tumor NMNAT-1/NAMPT/NRK1 dependence; active metabolite formation; IMPDH2 engagement; BBB penetration	Tumor burden and survival in orthotopic models; interaction with temozolomide	Exploits tumor NMNAT-1 activity rather than augmenting neural NMNAT-1	Requires tumor selectivity, toxicity and immune surveillance, resistance monitoring and clinical validation	Preclinical orthotopic GBM. [141]
